# Revisiting the Resazurin-Based Sensing of Cellular Viability: Widening the Application Horizon

**DOI:** 10.3390/bios12040196

**Published:** 2022-03-25

**Authors:** Darja Lavogina, Helen Lust, Maris-Johanna Tahk, Tõnis Laasfeld, Hans Vellama, Naila Nasirova, Markus Vardja, Kattri-Liis Eskla, Andres Salumets, Ago Rinken, Jana Jaal

**Affiliations:** 1 Institute of Clinical Medicine, University of Tartu, 50406 Tartu, Estonia; helen.lust@ut.ee (H.L.); andres.salumets@ki.se (A.S.); jana.jaal@kliinikum.ee (J.J.); 2 Institute of Chemistry, University of Tartu, 50411 Tartu, Estonia; maris-johanna.tahk@ut.ee (M.-J.T.); tonis.laasfeld@ut.ee (T.L.); naila.nasirova@ut.ee (N.N.); ago.rinken@ut.ee (A.R.); 3 Competence Centre on Health Technologies, 50411 Tartu, Estonia; 4 Department of Computer Science, University of Tartu, 51009 Tartu, Estonia; 5 Department of Physiology, Institute of Biomedicine and Translational Medicine, University of Tartu, 50411 Tartu, Estonia; hans.vellama@ut.ee (H.V.); kattri-liis.eskla@ut.ee (K.-L.E.); 6 Centre of Excellence for Genomics and Translational Medicine, University of Tartu, 51010 Tartu, Estonia; 7 Department of Radiotherapy and Oncological Therapy, Tartu University Hospital, 50406 Tartu, Estonia; markus.vardja@kliinikum.ee; 8 Division of Obstetrics and Gynaecology, Department of Clinical Science, Intervention and Technology (CLINTEC), Karolinska Institutet, 14186 Stockholm, Sweden

**Keywords:** resazurin, Alamar Blue, resorufin, fluorescence, absorbance, cell viability, hypoxia

## Abstract

Since 1991, the NAD(P)H-aided conversion of resazurin to fluorescent resorufin has been widely used to measure viability based on the metabolic activity in mammalian cell culture and primary cells. However, different research groups have used divergent assay protocols, scarcely reporting the systematic optimization of the assay. Here, we perform extensive studies to fine-tune the experimental protocols utilizing resazurin-based viability sensing. Specifically, we focus on (A) optimization of the assay dynamic range in individual cell lines for the correct measurement of cytostatic and cytotoxic properties of the compounds; (B) dependence of the dynamic range on the physical quantity detected (fluorescence intensity versus change of absorbance spectrum); (C) calibration of the assay for the correct interpretation of data measured in hypoxic conditions; and (D) possibilities for combining the resazurin assay with other methods including measurement of necrosis and apoptosis. We also demonstrate the enhanced precision and flexibility of the resazurin-based assay regarding the readout format and kinetic measurement mode as compared to the widely used analogous assay which utilizes tetrazolium dye MTT. The discussed assay optimization guidelines provide useful instructions for the beginners in the field and for the experienced scientists exploring new ways for measurement of cellular viability using resazurin.

## 1. Introduction

**Figure 1:**
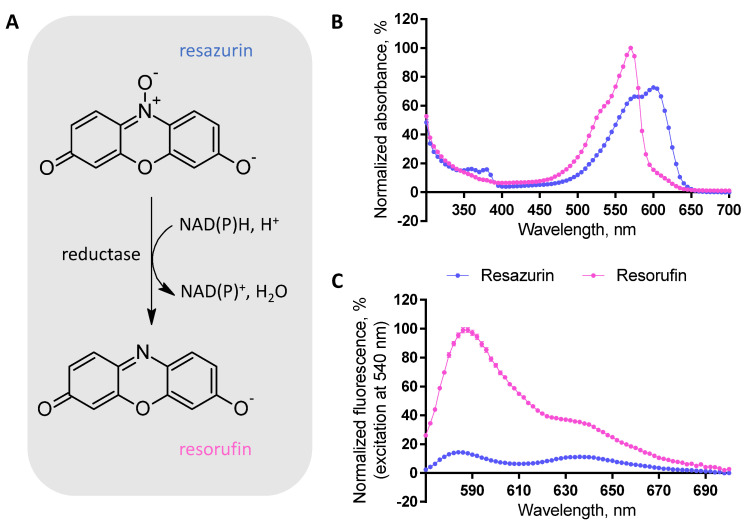
Structures and optical properties of resazurin and resorufin. (**A**) Schematic representation of the resazurin to resorufin conversion; enzymatic catalysis is required. (**B**,**C**) Absorbance and fluorescence spectra characteristics of the assay (100% corresponds to the maximal absorbance/emission intensity of resorufin and 0% to the absorbance/emission intensity of phosphate buffer within the measured wavelength range; average of triplicates ± SEM from a single representative experiment is shown). The resorufin spectra were registered after 2.5 h incubation of 50 μM resazurin with U-251 MG cells (96 h post-seeding, initial seeding density of 6250 cells/well) on the 96-well plate, while the resazurin spectra were registered after 3 h incubation of 50 μM resazurin on the 96-well plate in the absence of cells.

Resazurin (aka Alamar Blue) is a small-molecular-weight phenoxazin-3-one dye that can be reduced to resorufin by NADH, NADPH, FADH, or other biologically abundant reductive species in the presence of mitochondrial or cytoplasmic reductases [1] (Figure 1). At the physiological pH range of 7.4–7.5 [2,3], the conversion of resazurin to resorufin is associated with a shift in absorbance spectrum (resazurin: λ_max_ = 600 nm, resorufin: λ_max_ = 570 nm; Figure 1). Furthermore, upon excitation of the resorufin at the wavelengths corresponding to its absorbance maximum, fluorescence can be registered (λ_max_ = 590 nm); by contrast, resazurin is weakly fluorescent within the visible spectrum (Figure 1). The feasibility of colorimetric or fluorometric detection of the enzymatically catalyzed reduction of resazurin is combined with another attractive property of these dyes: despite carrying negative charge at pH 7.5 [3], both are cell plasma membrane-permeable.

It is thus not surprising that the resazurin-based assays have long been utilized for the monitoring of processes related to cellular respiration—which, in general terms, correlates with the overall viability status of various biological objects [1,4]. The initial reports (starting as early as 1939) focused onto the use of resazurin for testing of bacterial contamination in milk [5] or examination of antifungal susceptibility [6]; in 1991, resazurin was first applied for the assessment of motile sperm density [7], and within last 30 years, resazurin has been widely used as a viability sensor in multiple mammalian cell lines, primary cells, and even tumor spheroids [8].

In our research group, the routine application of resazurin for the detection of cellular viability was implemented five years ago. Since then, we have gained a wealth of data that is systematized here and analyzed regarding the pros and cons, application tips, issues, and notable aspects of the assay. In particular, we illustrate the optimization steps required for the high robustness of the assay, and address the risks related to combination of resazurin-based sensing with other fluorescent molecules (SYTOX Blue, doxorubicin, GFP-containing biosensor), hypoxia conditions, or radiation studies.

## 2. Materials and Methods

### 2.1. Apparatuses and Reagents

The immortalized human endometrial stromal cell line St-T1b was a kind gift from Dr. Martin Götte (University of Münster, Münster, Germany) [9]. All other cell lines used in this study were obtained from the American Type Culture Collection (ATCC; Manassas, VA, USA), the Leibniz Institute DSMZ—German Collection of Microorganisms and Cell Cultures GmbH (Braunschweig, Germany), or Invitrogen Life Technologies (Carlsbad, CA, USA). The detailed information on the sources of the commercially available cell lines and the suitable growth media is provided in Supplementary Table S1. Other solutions and growth medium components for the cell culture were obtained from the following sources: phosphate-buffered saline (PBS), L-glutamine, fetal bovine serum (FBS)—Sigma-Aldrich (Steinheim, Germany); insulin—Sigma-Aldrich (St. Louis, MO, USA); and a mixture of penicillin, streptomycin, and amphotericin B—Capricorn (Ebsdorfergrund, Germany).

The cells were grown at 37 °C in 5% CO_2_ humidified incubators (Sanyo; Osaka, Japan); unless otherwise indicated, normoxic conditions were used. For the viability assay, the initial number of cells was counted using TC-10 cell counter (Bio-Rad; Hercules, CA, USA). For the live imaging of cells transfected with the apoptosis sensor Casper3-GR, as well as for the resazurin-based dynamic range studies under normoxic and hypoxic conditions and for the dose–response studies of cytotoxic and cytostatic agents, the cells were seeded onto transparent 96-well clear flat bottom cell culture plates BioLite 130188 (Thermo Fischer Scientific; Rochester, NY, USA). Sterile centrifuge tubes from Nerbe (Winsen, Germany) or Capp (Nordhausen, Germany) were used for collection of cell suspension and preparing necessary dilutions.

For hypoxia studies, two different hypoxic chambers were used. In the first case, cultured cells were placed in the modular incubator chamber (Billups-Rothenberg Inc, MIC-101), a flow meter was attached to the unit, and the chamber was flushed with 20 L of gas mixture (1% O_2_, 5% CO_2_, 94% N_2_). The chamber was sealed and placed into the incubator (37 °C); the gas mixture was exchanged after every 24 h. In the second case, hypoxic gas mixture (1% O_2_, 5% CO_2_, 94% N_2_) was supplied by gas controller into the incubator of Cytation 5 multi-mode reader system equilibrated at 30 °C (BioTek; Winooski, VT, USA).

For the radiation studies, 96-well or 12-well clear flat bottom plates BioLite 130185 were used (Thermo Fischer Scientific; Rochester, NY, USA). For the transfer of cells to irradiation facility, gas-permeable adhesive moisture seals were used (Brooks Life Sciences, Wotton, Surrey, UK). The plates were positioned between the 2 slabs of solid water phantom (30 cm × 30 cm × 5 cm below, 30 cm × 30 cm × 1 cm above the plates) and irradiated with doses 2 Gy or 4 Gy (Gantry angle 0°, collimator angle 0°, field size 25 cm × 25 cm, source to surface distance 100 cm, dose rate 0.6 Gy/min). During the radiation studies, the cells were exposed to 6 MV X-rays (Varian Truebeam 2.5).

For microscopy studies, the cells were grown on 96-well Ibidi black μ-plates (ibidi GmbH, Gräfelfing, Germany). For fixation of cells, methanol was obtained from Honeywell (Riedel-de Haën™, Seelze, Germany). For preparation of washing solutions, PBS (supplemented with Ca^2+^, Mg^2+^) from Sigma-Aldrich (Steinheim, Germany) was used. The nuclear stain 4′,6-diamidino-2-phenylindole (DAPI) was obtained from Invitrogen (Eugene, OR, USA).

Resazurin, 3-(4,5-dimethylthiazol-2-yl)-2,5-diphenyltetrazolium bromide (MTT), and PBS for the viability assay (supplemented with Ca^2+^, Mg^2+^) were obtained from Sigma-Aldrich (St Louis, MO, USA). SYTOX Blue nucleic acid stain was obtained from Thermo Fischer Scientific (Rockford, IL, USA). The cytotoxic and cytostatic compounds were from the following sources: staurosporine—Cell Guidance Systems (Cambridge, UK); bortezomib, doxorubicin, and monomethyl auristatin E (MMAE)—TBD Biodiscovery (Tartu, Estonia); ARQ 092, MLN8237 (alisertib), VX-689, and MK-2206—Selleckchem (Munich, Germany); CX-4945—Synkinase (Shanghai, China); and paclitaxel (6 mg/mL solution)—Fresenius Kabi (Warsaw, Poland). The stock solutions of all compounds, except paclitaxel, were prepared in cell culture grade DMSO (AppliChem; Darmstadt, Germany) into low-binding microcentrifuge tubes (Eppendorf; Hamburg, Germany) and stored at −20 °C. For preparation of the formazan solubilization solution in the MTT assay, sodium dodecyl sulphate (SDS) from AppliChem (Darmstadt, Germany) and hydrochloric acid (HCl) from Honeywell (Riedel-de Haën™, Seelze, Germany) were used.

The fluorescence intensity and absorbance measurements were routinely carried out with Synergy NEO or Cytation 5 multi-mode readers (both from Biotek; Winooski, VT, USA). In one series of experiments, a Labexim Ledetect 96 microplate reader (Lengau, Austria) was used. Fluorescence microscopy was carried out with Cytation 5 multi-mode reader. The imaging of fixed cells was carried out with the Olympus (Tokyo, Japan) 20× air objective lens (LUCPLFLN, working distance 6.6, numerical aperture 0.45); for DAPI, 365 nm LED and DAPI filter block were used. The live imaging of cells transfected with Casper3-GR was carried out with the Olympus 4× air objective at 37 °C in the presence of 5% CO_2_. For excitation of GFP, 465 nm LED was used. Changes in FRET were measured by monitoring the emission using GFP and RFP filter blocks (525 nm and 593 nm, respectively). The imaging lasted for 23 h with 4 images taken per well at each time-point (ca. every 30 min), and automated focusing by GFP channel was used.

### 2.2. Resazurin Assay

The general protocol of the resazurin assay was published previously [10]; the experimental details are listed below.

#### 2.2.1. Dynamic Range Experiments

Two-fold dilution series of cell suspension in the growth medium (containing FBS) were carried out directly on the 12-well or 96-well flat-bottom plates. The cells were first grown for 24 h in the CO_2_ incubator at 37 °C under normoxic conditions and, in some studies, subsequently for further 24 h, 48 h, or 72 h in the CO_2_ incubator (30 °C or 37 °C) under normoxic or hypoxic conditions. In the case of 96-well plates, for 24 h experiments, the total volume of liquid per well was 100 μL, while for the prolonged experiments, the volume was increased to 200 μL; in the case of 12-well plates, the total volume of liquid per well was 1 mL. Afterwards, the growth medium was removed, the cells were rinsed with PBS, and solution of resazurin in PBS (supplemented with Ca^2+^ and Mg^2+^) was added (25 μM, 50 μM, or 100 μM). The plate was immediately transferred to the microplate reader (Biotek NEO or Cytation 5) and readings were taken at 30 °C or 37 °C in the kinetic mode (every 15 min for 2 h) using the following parameters: (A) fluorescence intensity— excitation at 540 nm, emission at 590 nm using a monochromator, top optics, gain 50, a slit width of 15 nm for NEO and 20 nm for Cytation 5; (B) absorbance—between 570 nm and 600 nm using a monochromator, and a read height of 8.5 mm. For experimental series with Labexim Ledetect 96, the plate was manually measured every 15 min (absorbance at 540 nm and 620 nm, using filters), and during the measurement, intervals were kept at pre-warmed using the Styrofoam box (30 °C).

#### 2.2.2. Radiation Studies

The cells were seeded onto the flat-bottom plates (96-well or 12-well) and grown in the usual growth medium in the CO_2_ incubator at 37 °C for 24 h. Next, the plates were sealed with the oxygen-permeable seals and transported to the irradiation facility. Non-irradiated plates were also transported to the irradiation facility but were kept in the linear accelerator’s control room. Irradiation was performed at room temperature.

Following the irradiation, the medium was exchanged (FBS-containing growth medium was used consistently) and the cells were further grown for 48 h (for 96-well plates) or 72 h (for 12-well plates). The viability measurements were subsequently carried out as described in Section 2.2.1.

#### 2.2.3. Experiments with Cytotoxic and Cytostatic Agents

The cells were seeded onto the flat-bottom plates (96-well or 12-well) and grown in the usual growth medium in the CO_2_ incubator at 37 °C for 24 h. Next, the medium was exchanged (dependent on the study, either usual growth medium containing FBS, or the FBS-deprived medium was used). The dilutions (three-fold, five-fold, or six-fold dilution series for dose–response studies) of chemicals in PBS were made on a separate 96-well plate or in centrifuge tubes and then added to the cell plates (volume ratio of ^1^/_4_ or ^1^/_5_, compared to the volume of medium). As a 100% viability control, either PBS alone or 0.1% DMSO in PBS was used. The cells were further grown for 24 h, 48 h, or 72 h in the CO_2_ incubator at 37 °C, and the measurements were carried out as described in Section 2.2.1.

#### 2.2.4. Measurement of Absorbance and Fluorescence Spectra

The spectra were recorded following the viability studies (as described in Section 2.2.1) at 30 °C or 37 °C using the following parameters: (A) fluorescence intensity—excitation at 540 (10) nm, emission scanned within the range of 570 to 700 nm or 580 to 700 nm (step of 2 nm) using a monochromator, top optics, gain 50, and a read height of 8.5 mm; (B) absorbance—within the range of 300 to 700 nm (step of 5 nm) using a monochromator.

### 2.3. Microscopy Assay

A two-fold dilution series of cell suspension in the growth medium (containing FBS) was carried out directly on the microscopy plates. The cells were first grown for 24 h in the CO_2_ incubator at 37 °C under normoxic conditions and subsequently for further 72 h in the CO_2_ incubator (30 °C or 37 °C) under normoxic or hypoxic conditions; the total volume of liquid per well was 200 μL. Afterwards, the growth medium was removed, the cells were rinsed with PBS, fixed with cold methanol and subjected to DAPI staining according to the previously published protocols [11]. The imaging settings were as follows: LED intensity 4, signal integration time 104 ms, and detector gain 5.

### 2.4. Studies Using the Apoptosis Sensor Casper3-GR

A previously reported version of the caspase-3 FRET-based biosensor Casper3-GR (originally purchased from Evrogen, Moscow, Russia) compatible with the baculovirus-based BacMam transduction system was used [12]. The transfection, further treatment, and imaging of HeLa cells was carried out according to the previously published protocols [12], except that the seeding density was 20,000 or 40,000 cells per well. In experiments comparing non-transfected and transfected cells, both types of cells were seeded onto the same experimental plate (half of the wells—non-transfected cells, half of the wells—transfected cells) and the whole plate was imaged using the same settings. After the imaging, the medium was removed, the cells were rinsed with PBS, and the viability measurements were carried out as described in Section 2.2.1.

### 2.5. Studies Using the Necrosis Sensor SYTOX Blue

The general protocol of the SYTOX Blue assay was published previously [10]. Briefly, the assay was carried out directly prior to the viability measurement; after removal of the growth medium from the cells, 1 µM SYTOX Blue solution in PBS (containing Ca^2+^ and Mg^2+^) was added. The plates were placed into a multimode reader, incubated for 10 min at 37 °C, and the fluorescence intensity was then measured (excitation at 430 nm, emission at 480 nm, monochromator, top optics, gain 90; area scan mode 5 × 5, read height 2.5 mm).

### 2.6. Studies Using the MTT Dye

The dilutions of cell suspension were made on the 96-well flat-bottom plates as described in Section 2.2.1; the volume of growth medium was 100 μL per well. The cells were grown for 48 h in the CO_2_ incubator at 37 °C under normoxic conditions. Next, to each well, 10 μL of the MTT solution in PBS (5 mg/mL) was added and the plates were further incubated in the CO_2_ incubator at 37 °C for 90 min or 180 min. Afterwards, 100 μL of the incubation solution was removed from each well and 100 μL of the formazan solubilization solution (10% SDS in 0.01 M HCl) was added. Following the 4 h incubation of the plates in the CO_2_ incubator at 37 °C, absorbance at 570 nm was measured as described in Section 2.2.1.

### 2.7. Data Analysis

All described measurements were performed as at least three independent experiments (N ≥ 3), with the exception of studies using the Labexim Ledetect 96 reader (N = 2), studies with variable resazurin concentration (N = 2), studies with a St-T1b cell line (N = 2), irradiation studies at various doses (N = 2 for each cell line), studies with variable passages of U-251 MG and T98-G cells (N = 2), and MTT assays (N = 2 for each incubation time with an MTT solution). For general data analysis, GraphPad Prism 6 (San Diego, CA, USA) and Excel 2016 (Microsoft Office 365; Redmond, WA, USA) were used.

The Z-factor of the resazurin-based viability assay for different cell lines, cell growth times, and detected optical characteristics (Supplementary Table S2) was calculated based on the data measured following 60 min (fluorescence intensity) or 90 min (absorbance ratio) incubation with 50 μM resazurin according to the Equation (1) (*σ_p_* and *σ_n_* stand for the standard deviation values of the positive and negative controls, respectively; *μ_p_* and *μ_n_* stand for the mean values of the positive and negative controls, respectively). As positive control, the highest cell number (non-treated or vehicle-treated) within the indicated dynamic range was used, and as a negative control, a well with zero seeded cells (incubated with resazurin solution for the same duration as the positive control). The Z-factor of the MTT assay was calculated analogously.

(1) $$Z=1-\frac{3\cdot \left(\sigma_{p}+\sigma_{n}\right)}{\left|\mu_{p}+\mu_{n}\right|}$$

For calculation of Z-factor, all available replicates from all independent experiments carried out at the identical conditions were pooled. In the case of experiments with primary cells, the data for each patients’ cells were first normalized (22 h or 22 h + 24 h incubation with 0.1% DMSO set as 100% viability control, and incubation with 10% DMSO set as 0% viability control) and the Z-factor was calculated based on the pooled normalized data (100% viability = positive control, 0% viability = negative control).

The dose–response curves of the cytotoxic and cytostatic agents were fitted to the three-parameter logarithmic equation, or to biphasic equation with the Hill slope values fixed at −1 and the fraction of the curve derived from the more potent phase fixed between 0 and 1.

The data analysis in the microscopy assay and the Casper3-GR assay was carried out in accordance with previously published procedures [11,12], respectively.

## 3. Results and Discussion

### 3.1. The Dynamic Range Concept: The Choice of the Detected Optical Characteristic, Resazurin Concentration, and Assay Time

As mentioned above, the resazurin-based viability assay can be carried out either with the colorimetric or fluorometric detection or both, depending on the availability of the suitable apparatuses and/or the properties of the studied biological system. To detect the fluorescence intensity, excitation is usually carried out within the range of wavelengths where absorbance of the forming resorufin is high, while the absorbance of remaining resazurin is relatively low (around 530–540 nm; see Figure 1 and Supplementary Figure S1). For absorbance detection, either an increase in absorbance at the wavelengths corresponding to resorufin maxima (540–570 nm) or a decrease in absorbance at the wavelengths corresponding to resazurin maximum (585–620 nm) can be monitored during the conversion of resazurin to resorufin. Alternatively, a ratiometric measurement format can be chosen where the change in absorbance ratio at two different wavelengths (one corresponding to resorufin and another corresponding to resazurin maximum) can be quantified; such ratiometric format augments the measurement window and enables partial elimination of minor pipetting mistakes or system interferences.

**Figure 2:**
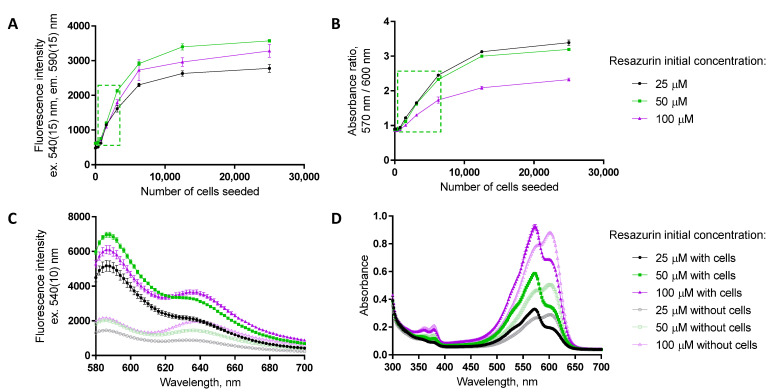
Optimization of the detected optical characteristic and resazurin concentration. (**A**,**B**) Dynamic range studies in T98-G cells, at 72 h post-seeding; initial concentration of resazurin indicated in the legend on the right (assay duration in the presence of resazurin: 2 h). The green dashed box indicates the linear dependence of the assay readout on the number of seeded cells for a 50 μM initial concentration of resazurin. (**C**,**D**) Fluorescence and absorbance spectra of resazurin solutions (initial concentration of resazurin indicated in the legend on the right) after 2 h assay duration in the absence or presence of T98-G cells (at 72 h post-seeding, initial seeding density of 3125 cells/well) on the 96-well plate. In each graph, average of duplicates ± SEM from a single representative experiment is shown.

The choice of readout needs to be accompanied with the optimization of (A) the dynamic range of the assay to ensure that the detected signal is indeed dependent on the number of viable cells (please see also Section 3.2) and (B) the resazurin concentration used to ensure the sufficient measurement window. Some examples of dependence of the assay dynamic range and measurement window on the resazurin concentration used (25, 50, or 100 μM at the beginning of the assay) are presented in Figure 2 and Supplementary Figure S1. For fluorescence intensity detection (excitation at 540 nm, emission at 590 nm), the measurement window is widest for the 50 μM resazurin. This probably reflects the depletion of resazurin by the high numbers of cells when 25 μM concentration is used, whereas in the case of 100 μM resazurin concentration, the excitation of the formed resorufin might be problematic due to interference from the remaining resazurin in solution. To detect the absorbance ratio (570 nm/600 nm), the measurement window becomes narrow at increasing concentrations of resazurin, as the remaining amount of non-consumed resazurin (Figure 2D) interferes with resorufin formed during the reaction. Overall, 50 μM starting concentration of resazurin seems optimal, at least in the case of a 96-well format where a 100–200 μL volume of resazurin solution per well is used.

Compared to the analogous assays utilizing tetrazolium dyes [13,14] (see also Section 3.8), or to the assays quantifying intracellular ATP [15], the resazurin-based assay has an important advantage: due to the cell membrane permeability and water solubility of the resorufin, there is no need to solubilize the product formed at the end of the reaction or to lyse the cells. This enables monitoring of the resazurin-to-resorufin conversion in time (Supplementary Figure S2), adding more flexibility to the assay and reducing the number of pipetting steps as well as reagent consumption.

The optimal assay duration is yet again dependent on the optical characteristic detected (Supplementary Figure S2), i.e., fluorometric measurements are more sensitive (when measuring the dynamic range, the top plateau is attained with a lower number of seeded cells), yet the ratiometric colorimetric measurements enable longer assay duration that might be beneficial if differences in cellular viability between different wells are quantified (see also Section 3.3 and Section 3.5). Furthermore, the ratiometric colorimetric format is generally more robust regarding the apparatus parameters (e.g., detector sensitivity), allowing easier pooling of data (Supplementary Figure S2A–D). Still, if a different pair of wavelengths is chosen (e.g., 540 nm/620 nm instead of 570 nm/600 nm), the dynamic range of the assay needs to be re-established (Supplementary Figure S2E,F).

### 3.2. Dynamic Range Dependence on the Cell Line, Time Post-Seeding, and Time in Culture

**Figure 3:**
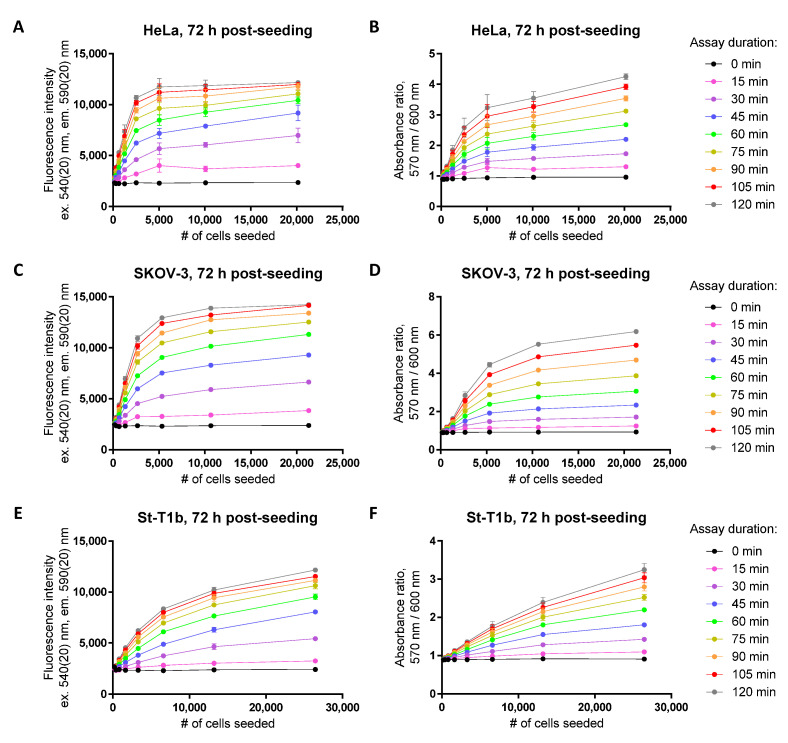
Establishment of the dynamic range in the resazurin assay for cervical cancer (HeLa; **A**,**B**), ovarian cancer (SKOV-3; **C**,**D**), or healthy immortalized endometrial stroma (**E**,**F**) cell lines at 72 h post-seeding. Detected optical characteristic: fluorescence intensity (excitation at 540 nm) in (**A**,**C**,**E**); absorbance ratio (570 nm/600 nm) in (**B**,**D**,**F**). Measurements were carried out at 50 μM resazurin (incubation times with resazurin indicated in the legend on the right). In each graph, the average of duplicates or triplicates ± SEM from a single representative experiment is shown.

**Figure 4:**
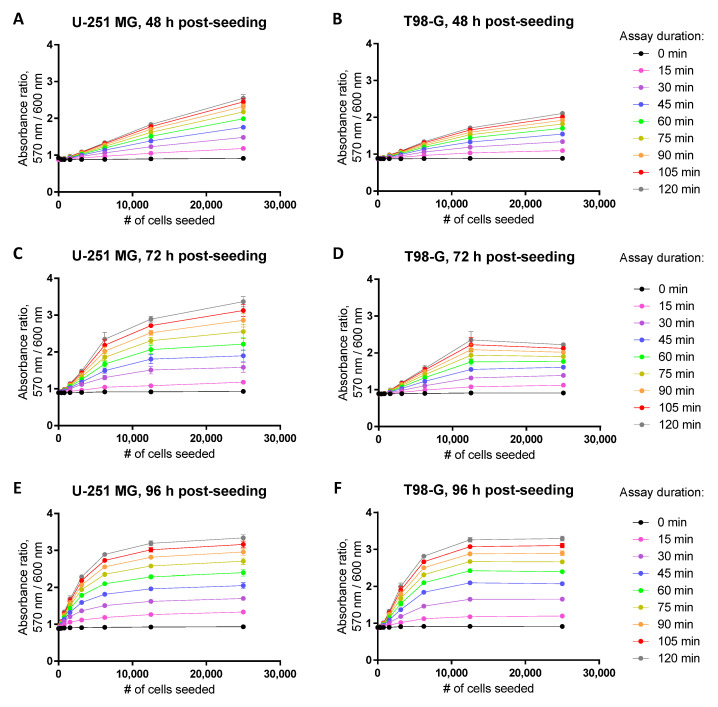
Establishment of the dynamic range in the resazurin assay for glioblastoma cell lines U-251 MG (**A**,**C**,**E**) or T98-G (**B**,**D**,**F**) at 48 h (**A**,**B**), 72 h (**C**,**D**), or 96 h (**E**,**F**) post-seeding. Detected optical characteristic: absorbance ratio (570 nm/600 nm). All experiments were carried out in the 96-well plate format at 50 μM resazurin (incubation times with resazurin indicated in the legend on the right). In each graph, an average of triplicates ± SEM from a single representative experiment is shown.

Because the size of the cells and the cellular metabolism rates vary in different cell lines, it is not surprising that the dynamic range of the resazurin-based assay is strongly dependent on the cell line. Figure 3 and Figure 4 and Supplementary Figure S3 illustrate the assessment of dynamic range for a total of nine different adherent cell lines (see also Supplementary Table S1 for details), among those eight originate from the cancerous tissues (glioblastoma, non-small cell lung cancer, prostate cancer, ovarian cancer, cervical cancer) and one originates from the healthy tissue (endometrium). Generally, cells that tend to look very large under the microscope (e.g., SKOV-3 or HCC-44) necessitate seeding of a fewer number of cells for the assay to remain within the dynamic range limits, which is probably related to the cells switching to a quiescent state following high surface confluency.

Naturally, the dynamic range of the assay will also become dependent on the time passed between the seeding of the cells and the measurement of viability, as the confluency of the well surface increases in time (Figure 4). The Supplementary Table S2 summarizes the suggested initial cell seeding densities (number of cells per well) for the resazurin-based viability assay in different cell lines, times post-seeding, and assay formats (regarding both the plate type and the detected optical characteristic). In cases where the prolonged treatments (viability measurement at 96 h post-seeding) are required (see Section 3.3), it is advisable to seed the cells on the 12-well rather than 96-well plate, despite a reduction in the experimental throughput. It should be noted that we did not carry out an additional optimization of the initial resazurin concentration for different plate formats; thus, further improvement in the assay robustness is possible.

To assess the performance of different assay formats in various cell lines for variable treatment durations, we calculated the Z-factor for the pooled data in identical treatments, using the highest cell number of the suggested dynamic range as a positive control and the resazurin signal in the absence of cells as a negative control (Supplementary Table S2). In general, assays with Z-factor values over 0.5 are considered very robust [16], and assays with Z-factor values between 0.4 and 0.5 are considered sufficiently reliable for semiquantitative studies [17]. For the resazurin assay, the Z-factor somewhat depended on the cell line and detected optical characteristic (due to the larger signal-to-background ratio in the fluorescence intensity measurements), converging to an average Z-factor value of 0.77 for fluorometric and 0.69 for colorimetric format (for calculation of the average Z-factor, only data for assays utilizing 96-well plates and normoxic conditions were used).

Furthermore, we explored how the repetitive passaging of cells affects the dynamic range of the assay. While the general guidelines do not recommend culturing of cells for more that 10–20 passages [18,19], in common practice, the culturing is frequently continued until the proliferation rate of cells decreases markedly. We measured the viability of two glioblastoma cell lines, U-251 MG and T98-G at 48 h or 72 h post-seeding when the cells were at the initial stages of culturing (3rd passage, P3), or after 3 months of culturing (30th passage, P30). Interestingly, we observed that the effect of culturing depended on the cell line (Supplementary Figure S4). For U-251MG, the apparent viability was consistently lower in P30 as compared to P3 cells, although the initial seeding density was identical. For T98-G, however, the apparent viability was consistently higher in P30 as compared to P3 cells. Although the observed effects can be affected by metabolic changes in cells during the culturing, those were consistent with our observations regarding the confluency of cells on the surface (data not shown). When the cells are grown in the same serum and regularly split using the same dilution factor, the U-251 MG cells tend to proliferate slower at increased passages, while T98-G cells proliferate faster (we did not monitor the cells further than 35th passage). Thus, changes in the proliferation rate during the passaging might depend on the cell line (even for lines with similar physiological background), complicating the interpretation of experiments performed at very different cell passage numbers within the same cell line—or, even more so, within different cell lines (see also Section 3.5).

### 3.3. Combination of Resazurin Assay with Irradiation Experiments

The experiments involving ionizing radiation serve as an example of application necessitating prolonged post-treatment protocols. Although DNA damage markers such as γ-H2AX are detectable within less than an hour after irradiation, the irradiated cells initiate DNA damage repair programs [20,21,22]; hence, the effect of ionizing radiation on the level of cellular viability is measurable following a significantly longer timescale. The ‘classical’ method for analysis of effect of ionizing radiation is the clonogenic assay which can be carried out even several weeks after the irradiation [23]. Here, we set out to explore whether the resazurin assay can be used for monitoring quicker responses on the level of cellular viability following irradiation, as shortening of assay times usually results in improved throughput.

Initially, we seeded the cells (two lung adenocarcinoma cell lines: A-549 and HCC-44; and two prostate cancer cell lines: DU 145 and PC-3) onto 96-well plates and irradiated some of the plates with various doses at 26 h post-seeding. The viability was then measured following 46–48 h of further incubation. To increase the temporal throughput, we simultaneously utilized two multiplate readers; hence, due to the necessity for the direct comparison of data between different plates (i.e., avoiding the need for in-plate normalization), we decided to carry out the resazurin assay only in the more robust colorimetric mode (see also Section 3.1). However, we found that in the case of this protocol, even upon irradiation at such a high dose of 10 Gy, the changes in the dynamic range of assay were minimal (Supplementary Figure S5). Based on the absorbance ratio readout, a slight reduction in viability was seen for the irradiated vs. non-irradiated plates in PC-3, and no remarkable changes were observed for DU 145 and HCC-44, whereas in the case of A-549, the viability was apparently increased.

**Figure 5:**
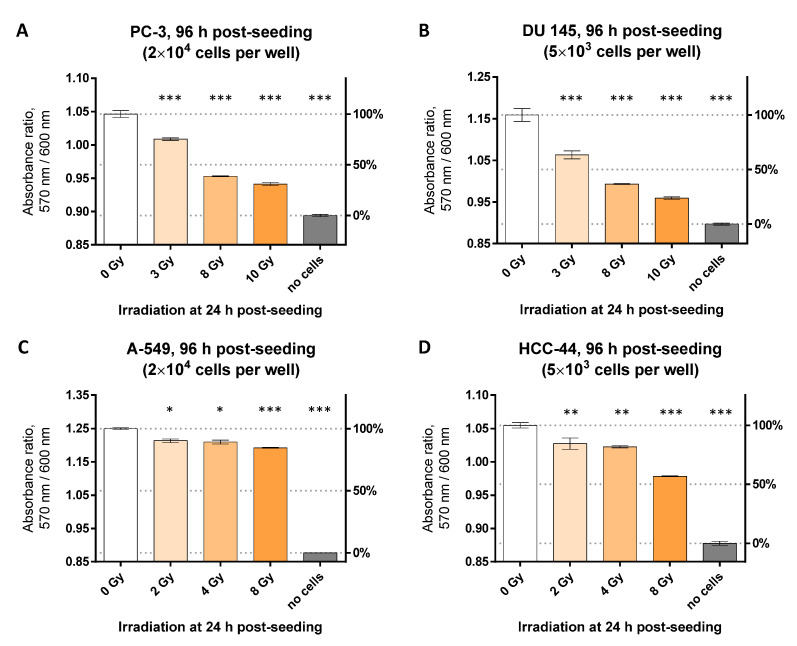
Resazurin assay following irradiation studies using different doses in various cell lines (12-well format). Irradiation of cells was carried out at 24 h post-seeding and measurement of viability was performed at 96 h post-seeding. Each graph presents pooled data (mean ± SEM) from 2 or more independent experiments. On the left Y-scale, the detected optical characteristic is shown (absorbance ratio, 570 nm/600 nm); on the right Y-scale, normalization of the pooled data is presented for easier interpretation (non-irradiated well with cells: 100% viability; non-irradiated well without cells: 0% viability). Measurements were carried out at 50 μM resazurin. (**A**) PC-3 cell line, incubation time with resazurin: 75 min; (**B**) DU 145 cell line, incubation time with resazurin: 60 min; (**C**) HCC-44 cell line; and (**D**) A-549 cell line, incubation time with resazurin: 90 min. The statistical significance of difference between the irradiated and non-irradiated wells was assessed using 1-way ANOVA with Dunnett’s test for multiple comparisons; *** indicates *p* ≤ 0.001, ** indicates *p* ≤ 0.01, * indicates *p* ≤ 0.05.

Thus, we decided to prolong the protocol by 24 h post-irradiation. To avoid achieving over-confluency of well surfaces, we switched from the 96-well to the 12-well plate format (see also Section 3.2), and measured the viability in the same cell lines after 72 h post-irradiation (at 96 h post-seeding). The results are shown in Figure 5 (comparison of absorbance ratio following irradiation of cells with different doses) and Supplementary Figure S6 (comparison of the dynamic range for non-irradiated cells and cells irradiated at 8 Gy). Briefly, for all cell lines, we saw a statistically significant (*p* < 0.05) decrease in viability even following irradiation at 3 Gy. The most dramatic dose-dependent decrease was observed for the DU 145 cell line, whereas the A-549 cell line was the least sensitive (within the timescale used). While further validation by the alternative assays could be beneficial to distinguish metabolism- vs. viability-associated effects, we have full confidence in applicability of the colorimetric resazurin assay for the assessment of radiation-induced viability decrease in the in vitro models.

### 3.4. Hypoxia and Hypothermia Studies: Validation of Applicability of Resazurin Assay Using Immunofluorescence

In recent decades, increased amount of attention has been paid to the conductance of cellular assays under reduced oxygen concentrations [24,25]. Given the fact that the normoxic conditions (over 20% of oxygen in the atmosphere) do not mirror the physiological reality on the tissue level—even though the layer of medium somewhat reduces the concentration of oxygen in the utmost proximity of the cultured cells, the experiments performed at hypoxic conditions are likely to exhibit increased physiological relevance of findings.

We thus set out to explore whether the resazurin assay can be used to measure the cell viability in hypoxia (1% of O_2_ in the gas mixture). For that, we utilized the glioblastoma cell lines U-251 MG and T98-G, and compared the dynamic range of the assay when performed at normoxic vs. hypoxic conditions. For both treatments, the cells were initially grown for 24 h at 37 °C in normoxic conditions following the seeding to let the cells attach to the 96-well plate; subsequently, the growth medium was exchanged, and some plates were transferred to the hypoxic conditions, whereas the control plates were left in normoxia. The cells were grown for 72 h prior to the viability measurement; to expand the horizon of the tried conditions, two independent experiments were carried out using 30 °C temperature (hypothermia) for the 72 h normoxic/hypoxic treatment, and three independent experiments were carried out using 37 °C temperature. The resazurin assay itself was carried out under normoxic conditions.

**Figure 6:**
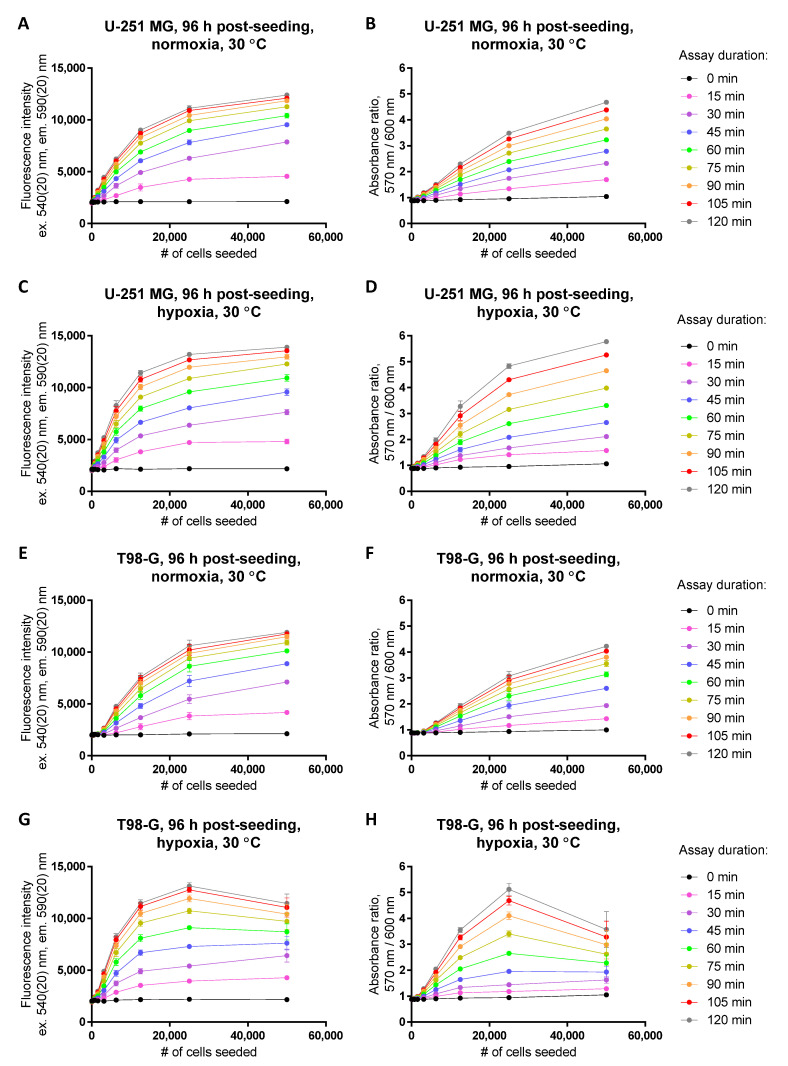
Establishment of the dynamic range in the resazurin assay following normoxic (**A**,**B**,**E**,**F**) vs. hypoxic treatment (**C**,**D**,**G**,**H**) at 30 °C in glioblastoma cell lines (**A**–**D**, U-251 MG; **E**–**H**, T98-G). For hypoxic treatment, cells were kept in hypoxic chamber (1% O_2_) for 72 h prior to measurement of viability (at 96 h post-seeding). Detected optical characteristic: fluorescence intensity (excitation at 540 nm) in (**A**,**C**,**E**,**G**); absorbance ratio (570 nm/600 nm) in (**B**,**D**,**F**,**H**). Measurements were carried out at 50 μM resazurin (incubation times with resazurin indicated in the legend on the right). In each graph, the average of duplicates or triplicates ± SEM from a single representative experiment is shown.

The results of the assay are summarized in Figure 6 (hypothermia) and Supplementary Figure S7 (37 °C). We observed that under hypoxic conditions, the extent of resazurin conversion to resorufin was overall increased as compared to the reaction depth in normoxic conditions at the identical cell seeding density. At low to medium cell seeding densities, such an apparent increase in cell viability was observable in both cell lines and at both temperatures. For high cell seeding densities, especially for the T98-G cell line following hypoxic treatment at 37 °C, a decline in resazurin conversion was observed as compared to the medium seeding density, probably indicating the problematic survival of cells in crowded environment with reduced oxygen availability. Therefore, the dynamic range of the resazurin assay (relative to the cell number) was narrower in hypoxia than in normoxia (Supplementary Table S2), indicating the need for precise optimization of the assay for application under hypoxic conditions. Such a narrow dynamic range also caused relatively large variability within independent experiments, which was reflected by the assay Z-factor values lower than 0.5 (Supplementary Table S2).

We next aimed to explore whether the apparent increase in resazurin conversion originates from the increased proliferation rate or from the increased metabolism rate of cancerous cells in hypoxia. Because the resazurin-to-resorufin conversion is dependent on the redox balance in cells [26], and due to the fact that resazurin can compete with oxygen during oxidation of NADH by the NADH oxidoreductase [27], we decided to use another assay for the parallel comparison of the obtained results. For that, we seeded the cells onto the 96-well microscopy plates, carried the treatment out for the resazurin assay, and then fixed the cells with methanol and stained nuclei with DAPI. The total nuclei count in 25 fields of view per well of a 96-well plate, which roughly corresponds to the number of cells (although multinucleated cells are not uncommon in U-251 MG or T98-G cells), was established using automated imaging and data analysis algorithm reported previously [11].

**Figure 7:**
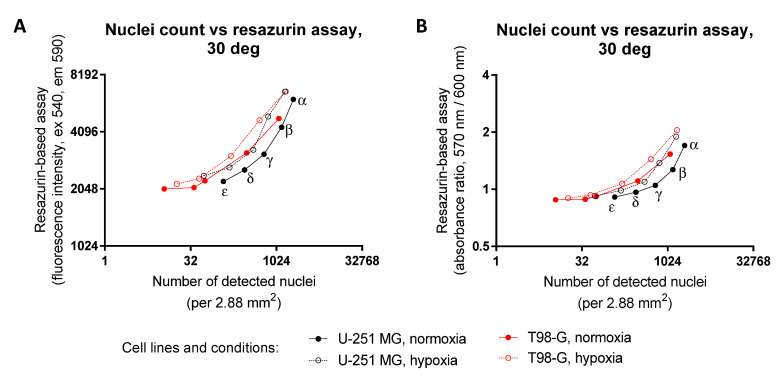
Comparison of data reflecting cellular viability measured using the detection of both metabolic activity and the number of nuclei following normoxic vs. hypoxic treatment of glioblastoma cell lines at 30 °C. Y-scale, the resazurin assay; X-scale, the microscopy assay; cell line names indicated under the graphs. Different initial seeding densities were used (for better visualization, indicated for the right-most curve only: α, 12,500 cells per well; β, 6250 cell per well; γ, 3130 cell per well; δ, 1560 cells per well; ε, 780 cells per well). For hypoxic treatment, cells were kept in the hypoxic chamber (1% O_2_) for 72 h prior to measuring the viability (at 96 h post-seeding). In the resazurin assay, measurements were carried out at 50 μM resazurin; fluorescence intensity (excitation at 540 nm) was detected in (**A**) (after 45 min incubation with resazurin) and absorbance ratio (570 nm/600 nm) in (**B**) (after 60 min incubation with resazurin). The nuclei were counted in 25 fields of view (dimensions of one field of view: 292 μm × 395 μm) using an automatic detection algorithm based on DAPI staining. Please note that the axes are logarithmic. In each graph, the average of triplicates from a single representative resazurin and a single representative microscopy experiment is shown; for better clarity, vertical and horizontal error bars were omitted.

The results of the microscopy assay following 72 h treatment of glioblastoma cells in normoxia or hypoxia at 30 °C or 37 °C are shown in the Supplementary Figure S8. The correlation between the readout of the resazurin assay and the microscopy assay for low to medium cell seeding densities and various treatments is presented in Figure 7 (30 °C) and Supplementary Figure S9 (37 °C). It can be noted that in the double logarithmic xy plots, the correlation curves corresponding to hypoxia treatment are systematically shifted to higher resazurin-to-resorufin conversion numbers, as compared to the correlation curves of normoxia (Figure 7, Supplementary Figure S9). This indicates increased conversion of resazurin to resorufin in hypoxia vs. normoxia for the same number of nuclei per well, thus showing that the apparent increase in the resazurin assay signals is related to the intensified use of resazurin as an oxidative agent, rather than the increased proliferation rate of glioblastoma cell lines in hypoxia. The graphs that are linear for the linear xy scale do not look linear in the double logarithmic xy plots; this originates from the non-zero y-intercept in the plotted dependence (i.e., some signals are measurable in the resazurin assay even when the number of nuclei detected by the microscopy assay is close to zero).

Overall, while our results show that the differences in the absolute values of signal in the resazurin assay following hypoxic vs. normoxic treatment of cells need to be cautiously interpreted, the assay is still potentially applicable for assessment of differences in viability within the same plate (e.g., following additional treatment with cytotoxic compounds) if the positive and negative controls are included on the same plate. The evaluation of the effects of cell viability modulators under hypoxic conditions represent a challenge in future studies; in the following sections of this manuscript, only studies at normoxic conditions will be addressed.

### 3.5. Resazurin Assay for Characterization of Cytotoxic and Cytostatic Effects of Compounds

**Figure 8:**
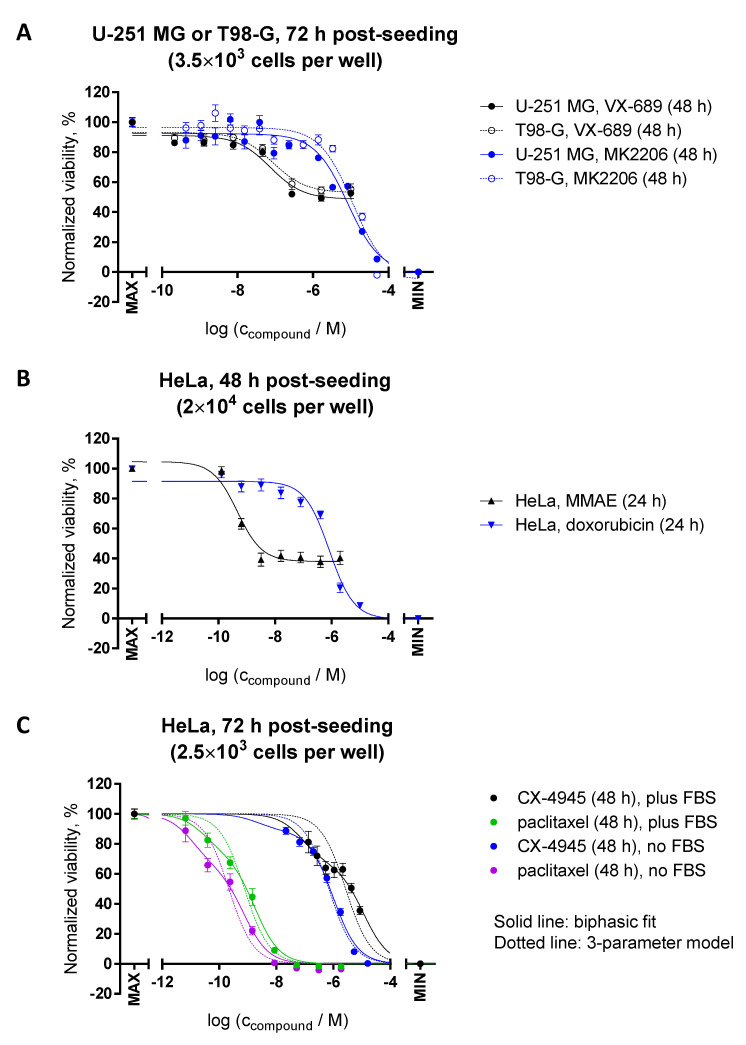
Resazurin assay for the assessment of cytotoxic and cytostatic effects of kinase inhibitors and chemotherapy agents. Glioblastoma (**A**) or HeLa (**B**,**C**) cells were treated with compounds for indicated duration (see legends on the right-hand side of the graphs) prior to measurement of viability (at 48 h or 72 h post-seeding). Measurements were carried out at 50 μM resazurin (incubation times with resazurin: within the linear range of the assay). In each graph, the average of duplicates ± SEM from at least two independent experiments is shown. Both fluorescence intensity (excitation at 540 nm) and absorbance ratio (570 nm/600 nm) were detected; for each independent experiment, the data were normalized separately for both optical characteristics and then pooled. For normalization, the following controls were used (MAX corresponds to 100% viability and MIN to 0% viability): (**A**,**C**) MAX = non-treated cells, MIN = well without cells; (**B**) MAX = treatment with 0.1% DMSO and MIN = treatment with 10% DMSO. For (**C**), the data were normalized separately for the cells grown in the presence or in the absence of FBS. The curves were fitted to the three-parameter logarithmic equations in the case of A and B (no additional restrictions were imposed during fitting), and to the biphasic (solid lines) or three-parameter logarithmic (dotted lines) equations for C (the top and bottom plateaus were fixed to 100% and 0%, and the Hill slope values to −1).

By far the most usual application of viability assays, including the resazurin assay, is monitoring the changes in viability of cells following treatments with compounds that modulate cellular survival and/or proliferation. Usually, such studies are carried out by measuring dose–response curves at different concentrations of the compound of interest. Importantly, the resazurin assay enables studies on the cytoprotective [28], cytotoxic, and cytostatic effects of compounds [8], though we will only focus on the two latter categories. Figure 8 illustrates some examples of the variations and trends in this assay format that might prove useful for a deeper understanding of the obtained data. Note that for each part of this figure, we pooled the normalized data from the fluorescence intensity and the absorbance ratio readouts that were measured within the same experiment but at different incubation times with resazurin (within the linear range of the corresponding formats), and thus can be considered as technical replicates (see also Supplementary Figure S10 for the examples illustrating notable consonance between the readouts).

By definition, the cytostatic agents inhibit proliferation of cells, but do not cause direct cytotoxicity [29]. In the resazurin assay, such ‘purely’ cytostatic compounds can be distinguished by the characteristic bottom plateau which does not reach the level of negative control (no cells), even at the elevated concentrations of a cytostatic compound (e.g., see dose–response curves for Aurora A inhibitor VX-689 in Figure 8A, or tubulin-binding agent MMAE in Figure 8B). Such differences in bottom plateau vs. negative control are best evident in the case of 24–48 h incubations with FBS present in the incubation medium, so that the initial population of the cells (which cannot divide in the presence of a cytostatic) still constitutes a significant fraction of the cells on the plate, but the cells are given a chance to at least double in number.

In reality, when the IC_50_ value of a compound of interest is relatively high, the feasibility of using higher doses is restricted (e.g., due to issues with solubility at high concentrations), which thus complicates the assessment of the bottom plateau location on the dose–response curve. Furthermore, different compounds can have a mixed profile exhibiting both cytostatic and cytotoxic effects—also dependent on the dosage, with cytotoxic effects usually occurring at higher concentrations [30]. In these cases, a comparison of experiments performed in the presence vs. absence of FBS in the incubation medium might prove useful. For compounds with preferred cytostatic effects, a reduction in the resazurin signal in the absence of FBS should be minimal, as the proliferation of cells is already strongly reduced at such conditions; the shift of IC_50_ values to the higher doses in the presence of FBS due to an increased cell number is thus more common for cytotoxic compounds. Figure 8C shows examples of dose–response curves for two compounds: casein kinase 2 inhibitor CX-4945 and a tubulin-binding agent paclitaxel (the normalization was carried out separately for the cells grown in the presence and in the absence of FBS, so that the viability of the 0.1% DMSO-treated control is equal to 100% in both cases). It is evident that both compounds show cytotoxic effects, whereas the shapes of the dose–response curves deviate remarkably from the simple three-parameter logarithmic model, thus indicating a complex mechanism of modulation of cell viability.

The choice of negative control for the normalization of data is another complex issue. Using the HeLa cell line, we demonstrated that the fluorescence intensity could be measured for a well containing resazurin, but no cells is essentially the same as for the well containing cells treated with 5 μM staurosporine, or a pan-kinase inhibitor with cytotoxic properties (Supplementary Figure S11A,B). Furthermore, the absorbance ratio was only slightly higher in the case of 5 μM staurosporine treatment. In this way, there are no significant optical aberrances originating from the presence of dead cells inside the well, although this statement needs further validation for cells with a high degree of granularity that might scatter the light. Furthermore, an analogous experiment in the PC3 cell line indicated no cellular death in the presence of staurosporine, consistently with the previously reported data [31] (Supplementary Figure S11C,D). This observation highlights the fact that application of high concentrations of well-known cytotoxic compounds as negative controls (especially in cases where the full dose–response curve is not measured) may not be a sufficiently robust approach in the case of assays with cell lines or primary cells that have not been studied in detail. Within the same set of experiments with HeLa and PC3, we also tried to utilize high concentrations of DMSO (10% by volume) that should result in the partial disintegration of the cell plasma membrane and thus serve as a relatively ubiquitous approach inducing cell death. We found that although 10% DMSO indeed dramatically reduced the viability of both cell lines (Supplementary Figure S11), the measured readout was still statistically significantly higher than the readout measured for the wells containing no cells. Still, in studies that require pooling of data from multiple experiments with limited amounts and/or altering seeding densities of previously uncharacterized cells (e.g., in studies with primary cells—see also Section 3.6), the 10% DMSO control is reasonable for normalization of data that might otherwise be prone to variation.

As already mentioned in the case of dynamic range studies (Section 3.2), an additional important aspect in the viability/proliferation assay is the number of cell passages carried out prior to the treatment. While it is important to avoid keeping the cell lines in culture for months, utilization of too early passages might also lead to high variation in experimental results. As an illustrative example, Supplementary Figure S12 shows changes in the effect of Aurora A inhibitor MLN-8237 used at 25 nM concentration when used in different early passages of the glioblastoma cell lines U-251 MG and T98-G (for each passage, the absorbance ratio measured for the MLN-8237 treated cells was normalized to the signals measured for the positive and negative controls). In studies at fixed concentrations of biologically active compounds where dose–response experiments are problematic due to the limited amount of compounds or cells, it is also advisable to monitor the effect of compounds at different treatment durations. For instance, in our chemosensitivity study with primary endometrial stromal cells (ESCs), we observed an initial apparent increase in viability in the presence of several compounds—which we could have interpreted as evidence of cytoprotective effects, unless another measurement after 24 h showed a decline in cell viability [10].

**Figure 9:**
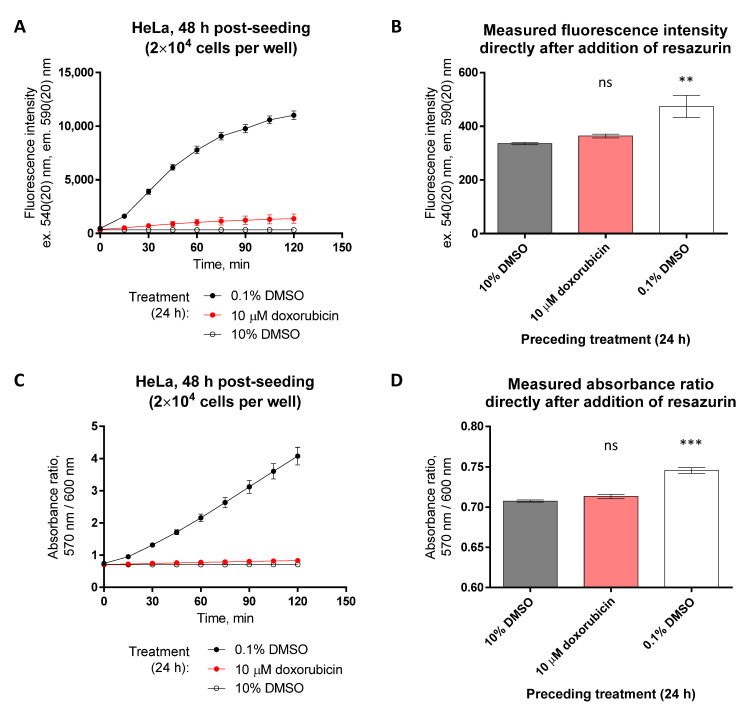
Pre-treatment of HeLa cells with fluorescent compound doxorubicin does not interfere with detection of resazurin fluorescence or absorbance ratio in solution. Cells were treated with compounds for 24 h prior to measurement of viability (at 48 h post-seeding). (**A**,**C**) Monitoring of change in fluorescence intensity (excitation at 540 nm) or absorbance ratio (570 nm/600 nm) during incubation of differently treated cells (conditions listed under the graphs) with 40 μM resazurin. (**B**,**D**) Values of fluorescence intensity or absorbance ratio directly following the addition of resazurin solution onto the cells (corresponding to the 0 min datapoint in graphs A and C, respectively). Please note the differences of the y-scale values in (**A**) vs. (**B)** and in (**C**) vs. (**D**). In each graph, an average of duplicates ± SEM from two independent experiments is shown. The statistical significance of difference between the zero-viability control (10% DMSO) and other treatments was assessed using 1-way ANOVA with Dunnett’s test for multiple comparisons; *** indicates *p* ≤ 0.001, ** indicates *p* ≤ 0.01, and ns indicates not significant. The significance of 0.1% DMSO vs. 10% DMSO treatment indicates the start of resazurin-to-resorufin conversion in viable cells within seconds following the addition of resazurin.

Although the straightforward interpretation of the resazurin assay data can be challenging, the assay has multiple beneficial characteristics. Apart from technical simplicity, flexibility regarding the measurement formats, and relatively low cost of resazurin, the assay is minimally sensitive to the presence or accumulation of other fluorescent compounds inside the cells, because the change in fluorescence intensity and absorbance ratio are measured in solution, not inside the cells. For instance, while the fluorescence intensity maximum of an anthracycline doxorubicin is in the same wavelength range as that of resorufin (see Supplementary Figure S13 and Figure 1C), the viability of cells treated with 10 μM doxorubicin can be feasibly monitored using 40 μM resazurin after the wash-out, and the putative interference of intracellular doxorubicin following the addition of resazurin to the cells is minimal (Figure 9). Such advantageous property means that the resazurin assay can be utilized in combination with other assays which utilize other fluorescent probes and sensors, as exemplified in Section 3.7.

### 3.6. Applicability of Resazurin Assay for Assessment of Viability of Primary Cells

Our experience with using the resazurin assay for viability studies in primary cells is currently limited with stromal cells isolated from the eutopic or ectopic endometrium (euESC and ecESC, respectively). In our previous study [10], we applied resazurin to explore the differences in chemosensitivity of these two types of samples, collected from a total of 11 patients, and pre-cultured for 5 passages. We utilized a protocol where the cells were seeded onto the 96-well plates, treated for 22 h with a variety of biologically active compounds or 0.1% DMSO as a control, followed by the first application of resazurin (40 μM) for 2 h. Next, we replaced resazurin solution with the sterile growth medium and kept the cells in the incubator for another 24 h, followed by the second round of measurement.

**Figure 10:**
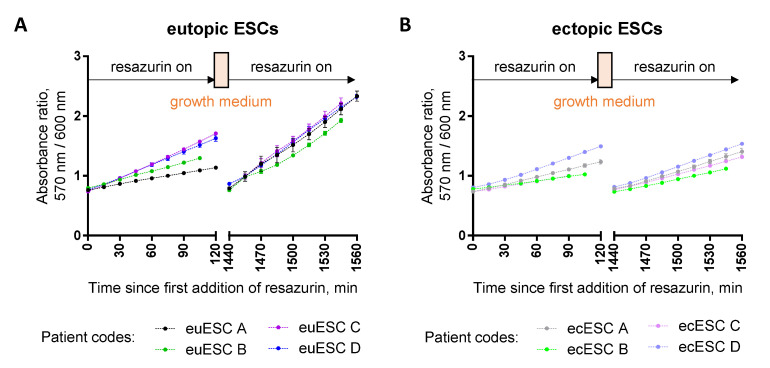
Resazurin assay in primary eutopic (**A**) and ectopic (**B**) endometrial stromal cells (ESCs) treated with 0.1% DMSO. The first measurement (120 min duration) was carried out at 24 h post-seeding; next, the cells were rinsed with PBS and kept in the growth medium in the CO_2_ incubator at 37 °C overnight, followed by the second measurement (also 120 min duration). Detected optical characteristic: absorbance ratio (570 nm/600 nm); measurements were carried out at 40 μM resazurin. In each graph, results are shown for ESCs isolated from the samples of four different patients; for each patient, an average of triplicates ± SEM is presented.

Despite the fact that both resazurin and resorufin penetrate the cell membrane easily and should not accumulate in cells following the wash-out, our initial concerns were connected to the potential toxicity of resazurin–resorufin [32,33] upon prolonged assay duration. As shown in Figure 10, which represents data regarding ESCs from four randomly chosen patients, at the beginning of the second measurement round, the absorbance ratio was reduced to the levels equal to those at the beginning of the first round, indicating the efficient removal of resorufin from the system. Furthermore, in euESCs, the absorbance ratio at the end of the second round was consistently higher than at the end of the first round, indicating proliferation of cells during the 24 h pause (Figure 10A). For ecESCs, however, the absorbance ratio values at the end of the first and the second round were rather comparable (Figure 10B). This might reflect differences in the initial number of seeded cells (ectopic biopsies tend to be smaller than eutopic ones), but also differences in metabolism, as our initial study pointed to the increased sensitivity of ecESCs (as compared to euESCs) to the agents acting on intracellular redox balance [10]. Interestingly, other groups have reported that resazurin toxicity is more pronounced in cancerous cells compared to healthy cells [34]. In this way, although we were limited regarding the available number of cells to conduct further experiments (e.g., a 48 h consecutive treatment control omitting the first round of assay), there was no sign of decrease in viability either for euESCs or ecESCs.

Due to the remarkable inter-patient variation characteristic to the primary cells, for calculation of the assay Z-factor, we needed to normalize the data for each patient prior to the pooling (treatment with 0.1% DMSO = 100% viability, treatment with 10% DMSO = 0% viability). For the pooled normalized data, the following Z-factor values were obtained: first round of measurement—0.64 (euESC, 11 patients) or 0.65 (ecESC, 11 patients); second round of measurement—0.60 (euESC, 10 patients) or 0.70 (ecESC, 10 patients). In the case of one patient’s ESCs, the second round of measurement was not performed, so no data were available. Overall, these data point to the suitability of the resazurin assay for experiments in primary cells, even in the case of more complex treatment protocols.

### 3.7. Combination of Resazurin Assay with Assays Utilizing Casper3-GR Biosensor and SYTOX Blue Dye

The resazurin assay measures changes in the redox metabolism that mirror a decrease in the cellular proliferation/viability, yet do not distinguish between the types of cellular death. We explored whether resazurin can be used in combination with the genetically encoded Casper3-GR biosensor for apoptosis studies, or whether chemical probe SYTOX Blue can enable studies of necrosis or late stages of apoptosis. Here, we present data for HeLa cells, although we have confirmed that similar strategies can be utilized also in other cell lines (PC-3, HEK293T—manuscript in preparation) and primary cells [10].

The Casper3-GR biosensor was originally developed by Evrogen (Moscow, Russia) and consists of a FRET pair of fluorescent proteins (TagGFP-TagRFP), flanking an amino acid sequence that can be selectively recognized by caspase-3 [35]. The apoptosis-initiated intracellular rise in caspase-3 levels triggers proteolytic cleavage of the sensor and a reduction in FRET efficiency, which can be monitored in real time using live microscopy in transfected cells [12]. Usually, monitoring Casper-3 signal changes starts immediately after apoptosis-inducing compounds are introduced, and lasts for 20 h or longer [12]. On such a timescale, parallel treatment of cells with resazurin could cause cellular toxicity; therefore, we applied resazurin following the live microscopy experiment. To check whether introduction of biosensors alters the chemosensitivity of HeLa cells, we also seeded non-transfected cells onto the same microscopy plate. The wells containing non-transfected control treatments were also imaged to minimize risk of artefacts originating from the possible cell damage upon prolonged irradiation with visible light.

The results of the resazurin assay in both transfected and non-transfected cells, as well as the results of the apoptosis assay, are presented in Table 1 and Supplementary Figure S14. At the lower seeding density used (2 × 10^4^ cells per well), the logarithms of IC_50_ values of both apoptosis-inducing compounds, staurosporine (broad-spectrum protein kinase inhibitor), and bortezomib (26S proteasome inhibitor) are within uncertainty limits for transfected and non-transfected cells. This indicates that the presence of Casper3-GR does not interfere with the resazurin assay. The IC_50_ values reported by Casper3-GR itself are remarkably lower, which might be explained by two aspects. First, the assays utilize different readouts—the live microscopy quantifies changes in individual cells [12], which may be affected more by the single events with dramatic FRET changes, whereas the resazurin reports on the averaged data for the well which are not as influenced by the outliers. Second, the cellular events measured by biosensor (i.e., activation of caspase-3 at the onset of apoptotic cascade) occur prior to the events measured by resazurin (reduction in cellular respiration associated with later stages of apoptosis), so there is an inherent delay time.

At a higher seeding density (4 × 10^4^ cells per well), the dose–response curves of both compounds are shifted to higher values in the resazurin assay (i.e., the apparent efficacy of cytotoxic compounds decreases). Such high seeding density might be beneficial for the Casper3-GR assay in order to collect sufficient statistics; however, in the case of the resazurin assay, this seeding density is far outside of linear range (Supplementary Table S2). This illustrates the fact that if both assays need to be utilized, a sensible compromise needs to be achieved regarding the parameters of either.

The principle behind the SYTOX Blue assay utilizes cell membrane-impermeable dye that can only enter cells with compromised integrity of plasma membrane. In such cells, the dye intercalates into DNA and forms a fluorescent complex [36]. Moreover, the intensity of the SYTOX Blue emission is thus proportional to the population of necrotic or late-apoptotic cells in the sample. Because the excitation wavelength of SYTOX Blue is quite low (maximum at 430 nm [37]), this assay can be easily affected by the autofluorescence originating from the buffer components, measurement plate plastics, etc. Furthermore, the necrotic cells with the most intense SYTOX Blue signal are poorly attached to the plate and can be accidentally discarded in repetitive washing procedures. On the other hand, the assay duration is relatively short (less than 30 min), as internalization of the dye through ruptured membranes and formation of DNA complex occurs rapidly [37]. Thus, following the 24 h treatment of HeLa cells with staurosporine (0.1 μM, 1 μM, or 5 μM) or DMSO (0.1%, 10%), we first performed necrosis or late apoptosis measurements using SYTOX Blue; however, to some wells, instead of 1 μM SYTOX Blue solution, only PBS was added. Next, we removed SYTOX Blue from the plate and carried out the resazurin assay and compared the fluorescence intensity and absorbance ratio readouts between the wells which were incubated with SYTOX Blue vs. PBS control for 20 min prior to the application of resazurin.

**Figure 11:**
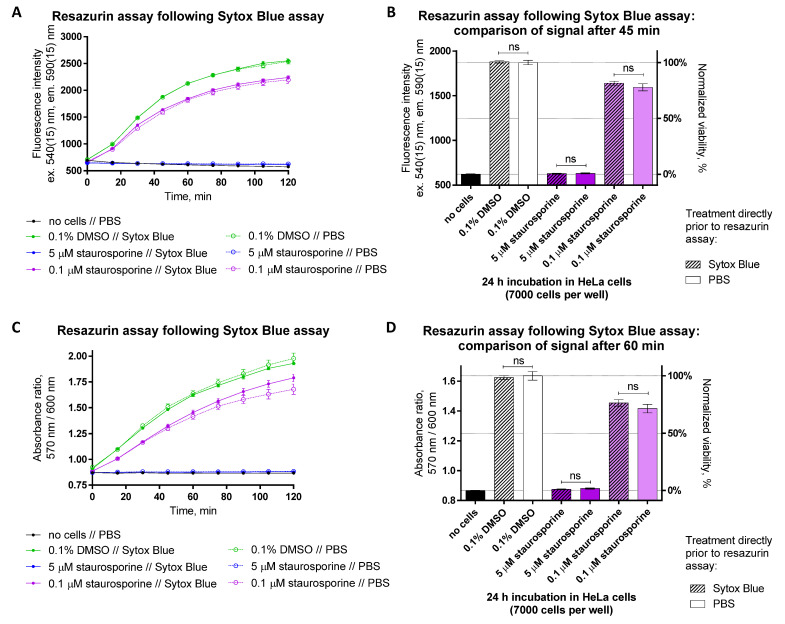
Resazurin assay can be combined with the SYTOX Blue assay assessing necrosis or late apoptosis in HeLa cells. Cells were treated with compounds (conditions indicated below graphs) for 24 h prior to measurement of necrosis or late apoptosis (data sets or columns marked as ‘SYTOX Blue’) and subsequent viability (at 48 h post-seeding). For assessing the impact of SYTOX Blue on the resazurin assay, some wells were treated with PBS only (not SYTOX solution in PBS) prior to the viability assay (data sets or columns marked as ‘PBS’). (**A**,**C**) Monitoring of change in fluorescence intensity (excitation at 540 nm) or absorbance ratio (570 nm/600 nm) during incubation of differently treated cells (conditions listed under the graphs) with 50 μM resazurin. (**B**,**D**) Values of fluorescence intensity or absorbance ratio during incubation of cells with resazurin at the time corresponding to the end of linear range shown in graphs (**A**,**C**), respectively (45 min for fluorescence intensity readout and 60 min for absorbance ratio readout). In each graph, an average of 2 or more replicates ± SEM from three independent experiments is shown. In (**B**,**D**), the statistical significance of pairwise comparisons between the signals measured for identical treatments incubated with SYTOX Blue or PBS prior to the resazurin assay was established using unpaired two-tailed *t*-test (95% confidence level) with Welch’s correction: ns stands for not significant.

The results of the SYTOX Blue assay are shown in Supplementary Figure S15. Expectedly, cells treated with staurosporine or 10% DMSO (causes permeabilization of plasma membrane) feature an elevated SYTOX Blue signal. The time course of resazurin reduction in differently treated cells is illustrated in Figure 11. Furthermore, no significant difference was found between the cells that had undergone incubation with SYTOX Blue and the cells otherwise treated with the same compounds yet incubated with PBS only prior to application of resazurin. Overall, because the signal measured using SYTOX Blue originates from the cells with compromised viability, whereas the signal measured using resazurin originates from the metabolically active cells, two distinct populations of major ‘analytes’ are formed, minimizing the risk for the unwanted crosstalk between the two assays.

### 3.8. Comparison of the Resazurin Assay with MTT Assay

Finally, we aimed to compare the resazurin-based assay with an analogous assay utilizing the tetrazolium dye MTT. The application of MTT as a sensor is also based on the NAD(P)H-aided reduction of the dye catalyzed by reductases in the mitochondria or in the cytosol of metabolically active cells [13,15]. However, different from the resazurin–resorufin conversion, the product formed after MTT reduction (formazan) is insoluble. Therefore, a separate solubilization step is required at the end of the MTT assay, which adds technical complexity. Organic solvents such as DMSO or N,N-dimethyl formamide affect the polystyrene-based measurement plates and are thus not compatible with the typical cell culture plastic, whereas the solubilization solutions using detergents (such as sodium dodecyl sulphate, SDS) necessitate prolonged solubilization steps (thus increasing the assay time) and decrease measurement precision due to foaming. Furthermore, the conversion of MTT to formazan cannot be monitored in an online regime, which reduces the assay flexibility. A more detailed comparison of the technical characteristics of the resazurin- and MTT-based assays is provided under the Supplementary Table S3.

To provide experimental insight into the comparison of two assays, we performed two pilot experiments applying the MTT assay in four cell lines that are routinely used in our ongoing projects: two glioblastoma cell lines (U-251 MG and T98-G) and two non-small-cell lung cancer cell lines (A-549 and HCC-44). A two-fold dilution series of cells was made for each cell line on the 96-well plates and the cells were grown for 48 h prior to the assay. To avoid the extensive optimization procedures for the MTT assay, we used the protocols recommended in literature [15,38]. The MTT final concentration during incubation with cells was 0.5 mg/mL, and two incubation times were used (90 min and 180 min). For solubilization, we used 10% SDS solution in 0.01 M HCl; the solubilization solution was applied for 4 h prior to measuring the absorbance at 570 nm. As a control, we carried out the resazurin assay with the similarly seeded cells.

The pooled results of two independent experiments are presented in the Supplementary Figure S16 (dynamic range assessment for the MTT assay and the colorimetric readout mode of the resazurin assay) and Supplementary Figure S17 (correlation between the data measured using resazurin vs. MTT at the same initial cell seeding density). Overall, for all cell lines measured, the measurement window of the MTT assay was comparable to that of the colorimetric mode of the resazurin assay. For the 90 min incubation with either sensor, the maximal fold change (i.e., ratio of signals measured in the wells with 25,000 cells versus the wells with no cells) was in the range of 2.71–3.74 for MTT or 2.49–2.82 for resazurin. In the case of the maximal assay duration, the corresponding values were in the range of 2.81–3.82 for MTT (3 h incubation) or 2.55–3.28 for resazurin (2 h incubation). Interestingly, while in the resazurin assay, the measurement window was wider for glioblastoma cell lines in the MTT assay, the measurement window was wider for the lung cancer cell lines (Supplementary Figure S16). This indicates that the two assays might have different preferences regarding the pools of reductases and NAD(P)H available in the cells.

Still, the dynamic range (i.e., the range of the seeding densities where the signal is proportional to the seeded cell number) was narrower in the MTT assay than in the resazurin assay, as especially evident from the curvature of the correlation-depicting graphs in Supplementary Figure S17A. The increase in the incubation time with MTT from 90 min to 180 min further decreased the dynamic range of the assay. Furthermore, the measurement uncertainty for the replicates was larger in the MTT assay. Although further optimization of the MTT assay might be possible, under the conditions tested in these pilot experiments, the Z-factor of the assay was found to be below 0.5 (in the range of 0.35–0.47) for three out of four cell lines. For HCC-44, the Z-factor value was 0.73. For comparison, the Z-factor of the resazurin assay calculated on the basis of the same two experiments ranged from 0.53 to 0.78. Thus, based on the trends observed in this brief demonstration, and taking into consideration the fact that the MTT assay yields remarkably less data per run while being more time-, reagent-, and plastic-consuming (Supplementary Table S3), we conclude that the performance of the resazurin assay is clearly superior.

## 4. Conclusions

The resazurin-based viability assay has been a ‘working horse’ in the field of cytotoxicity studies for the last two decades due to a wide horizon of advantageous properties:

- The working principle underlying the assay is intuitively clear;
- The assay does not require highly specialized apparatuses or highly qualified workforce. In fact, a smartphone camera might be sufficient for the tasks that require semiquantitative answers [39];
- Even when used for the demandingly quantitative purposes, the assay is relatively cheap. The routine costs of the cell maintenance in culture might easily outweigh the assay costs (resazurin powder, PBS, multi-well plates, and maintenance of a multiplate reader);
- There is flexibility in the readout modes, the reaction volumes, the duration, the nature of treatment, and other parameters;
- The measurements are carried out in the bulk of the solution covering the cells, thus being minimally affected by the intracellular presence of the potentially interfering fluorescent substances (e.g., biosensors, other chemical probes, or cytotoxic agents);
- Based on observations in all cell lines and primary cells tested so far, the application of resazurin as a sensor does not require any auxiliary reagents that might interfere with chemosensitivity of cells.

Still, there are several aspects that need to be considered to ensure the adequate application of resazurin for assessment of cellular viability:

- The dynamic range of the assay (regarding the number of seeded cells, the initial concentration of resazurin, the type of readout, and the assay duration) needs to be optimized for each cell line, treatment type, treatment duration, plate type, and apparatus used. For successful combination of the resazurin assay with other methods (e.g., apoptosis measurements), the set of optimized parameters should be compatible with requirements of both assays;
- Given that the resazurin assay directly measures metabolism rather than viability of cells, in cases where the treatment of cells involves substances or conditions which affect the redox balance, the results require cautious interpretation, application of a set proper treatment controls, and, whenever possible, methods based on alternative readouts (e.g., counting of cells using microscopy techniques).

The studies described in this manuscript provide the initial instructions for assay optimization. (The guidelines for the seeding densities in different cell lines are listed under the Supplementary Table S2, and the suggested experimental procedures for the optimization of the assay linear range and assessment of cytotoxicity of compounds of interest are provided in the Supplementary Protocols S1 and S2, respectively.) We hope that these instructions will be equally useful for the beginners in the field and the experienced scientists exploring new ways to exploit Alamar Blue for the measurement of cellular viability.

## Figures and Tables

**Table 1:** Comparison of logarithms of IC_50_ values ± SEM measured for staurosporine or bortezomib in the HeLa cell line using different assay formats (N = 3 for each type of assay).

Transfection Status ^1^	Number of Seeded Cells Per Well	log (IC_50_/M): Resazurin Assay		log (IC_50_/M): Apoptosis Assay ^2^	
		Staurosporine	Bortezomib	Staurosporine	Bortezomib
NT	2 × 10^4^	−7.7 ± 0.2	−7.3 ± 0.2	NM	NM
Casper3-GR	4 × 10^4^	−7.6 ± 0.2	−7.4 ± 0.2	−8.13 ± 0.08	−7.9 ± 0.2
Casper3-GR	4 × 10^4^	−6.8 ± 0.6	−7.32 ± 0.03	−8.7 ± 0.2	−7.6 ± 0.2

24 h treatment with compounds was carried out. ^1^ NT indicates not transfected (the imaging was still performed). ^2^ NM, not measured due to the absence of apoptosis sensor in cells.

## Data Availability

The trained model for the microscopy data analysis and the Aparecium 2.0 software suggested for the batch processing are available at https://gpcr.ut.ee/aparecium.html (accessed on 25 February 2022). The GraphPad Prism files corresponding to Figure 1, Figure 2, Figure 3, Figure 4, Figure 5, Figure 6, Figure 7, Figure 8, Figure 9, Figure 10 and Figure 11 and Supplementary Figures S1–S17 are publicly available via FigShare repository: https://figshare.com/s/e67a9ba0e38bda120aaa (accessed on 25 February 2022).

## Supplementary Materials

The following supporting information can be downloaded at: https://www.mdpi.com/article/10.3390/bios12040196/s1. Table S1: Sources of the cell lines used in the study and the corresponding growth media. Table S2: Suggested initial cell seeding densities (number of cells per well) for the resazurin-based viability assay in different cell lines. Table S3: Comparison of the technical characteristics of resazurin assay and 3-(4,5-dimethylthiazol-2-yl)-2,5-diphenyltetrazolium bromide (MTT) assay. Figure S1: Fold change of resazurin spectra in the presence vs. absence of cells. Figure S2: Example of absolute scale differences in detecting a fluorescence or absorbance ratio during the establishment of a dynamic range in the resazurin assay using different apparatuses. Figure S3: Establishment of the dynamic range in the resazurin assay for prostate cancer or lung adenocarcinoma cell lines at 72 h post-seeding. Figure S4: Establishment of the dynamic range in the resazurin assay for different passages of glioblastoma cell lines U-251 MG or T98-G at 48 h or at 72 h post-seeding. Figure S5: Establishment of the dynamic range in the resazurin assay for prostate cancer or lung adenocarcinoma cell lines following irradiation at 10 Gy (96-well format). Figure S6: Establishment of the dynamic range in the resazurin assay for prostate cancer or lung adenocarcinoma cell lines following irradiation at 8 Gy (12-well format). Figure S7: Establishment of the dynamic range in the resazurin assay following normoxic vs. hypoxic treatment at 37 °C for glioblastoma cell lines. Figure S8: Detection of the number of nuclei using the microscopy assay following normoxic vs. hypoxic treatment of glioblastoma cell lines at different seeding densities and temperatures. Figure S9: Comparison of data reflecting cellular viability measured using the detection of metabolic activity and detection of the number of nuclei following normoxic vs. hypoxic treatment of glioblastoma cell lines at 37 °C. Figure S10: Comparison of the dose–response curves measured using different readouts for a cytotoxic or a cytostatic compound. Figure S11: Resazurin assay in HeLa or PC3 cell lines: comparison of different negative controls. Figure S12: Effect of the Aurora A inhibitor MLN-8237 on the viability of glioblastoma cell lines dependent on the passage number of the latter. Figure S13: Fluorescence spectrum of HeLa cells treated with doxorubicin. Figure S14: The results of the resazurin assay are dependent on the transfection of cells with the Casper3-GR biosensor. Figure S15: Sytox Blue assay results in HeLa cells following 24 h treatment with staurosporine or DMSO. Figure S16: Comparison of the dynamic range in the resazurin assay and the MTT assay. Figure S17: Correlation between the data measured using the resazurin assay and MTT assay. Protocol S1: Establishment of the dynamic range in the resazurin assay. Protocol S2: Application of the resazurin assay for assessment of the cytotoxic properties of the compounds of interest.
