# Supplementary material for: Revisiting the Resazurin-Based Sensing of Cellular Viability: Widening the Application Horizon
Source: Biosensors (Basel). 2022 Mar 25;12(4):196. doi: 10.3390/bios12040196 (PMC9032648; doi:10.3390/bios12040196)
Supplement: Supplementary file 1 [file biosensors-12-00196-s001.zip › biosensors-1635664-supplementary.pdf]

biosensors

Supplementary Information

Revisiting the Resazurin-Based Sensing of Cellular Viability: Widening the Application Horizon

Darja Lavogina [1,2,3,*], Helen Lust [1], Maris-Johanna Tahk [2], Tõnis Laasfeld [2,4], Hans Vellama [5,6], Naila Nasirova [2], Markus Vardja [7], Kattri-Liis Eskla [5,6], Andres Salumets [1,3,8], Ago Rinken [2] and Jana Jaal [1,7]

[1] Institute of Clinical Medicine, University of Tartu, 50406 Tartu, Estonia; helen.lust@ut.ee (H.L.); andres.salumets@ki.se (A.S.); jana.jaal@kliinikum.ee (J.J.)
[2] Institute of Chemistry, University of Tartu, 50411 Tartu, Estonia; maris-johanna.tahk@ut.ee (M.-J.T.); tonis.laasfeld@ut.ee (T.L.); naila.nasirova@ut.ee (N.N.); ago.rinken@ut.ee (A.R.)
[3] Competence Centre on Health Technologies, 50411 Tartu, Estonia
[4] Department of Computer Science, University of Tartu, 51009 Tartu, Estonia
[5] Department of Physiology, Institute of Biomedicine and Translational Medicine, University of Tartu, 50411 Tartu, Estonia; hans.vellama@ut.ee (H.V.); kattri-liis.eskla@ut.ee (K.-L.E.)
[6] Centre of Excellence for Genomics and Translational Medicine, University of Tartu, 51010 Tartu, Estonia
[7] Department of Radiotherapy and Oncological Therapy, Tartu University Hospital, 50406 Tartu, Estonia; markus.vardja@kliinikum.ee
[8] Division of Obstetrics and Gynaecology, Department of Clinical Science, Intervention and Technology (CLINTEC), Karolinska Institutet, 14186 Stockholm, Sweden
* Correspondence: darja.lavogina@ut.ee; Tel.: +372-737-5296

Table S1. Sources of the cell lines used in the study and the corresponding growth media.

Cell line	Description of cell line	Cell line source	Growth medium [a]	Growth medium source
**A-549**	Human non-small cell lung adenocarcinoma	DSMZ	Dulbecco's Modified Eagle's medium (DMEM) High Glucose	Sigma-Aldrich (Steinheim, Germany)
**DU 145**	Human prostate carcinoma	DSMZ	DMEM High Glucose	Sigma-Aldrich (Steinheim, Germany)
**HCC-44**	Human non-small cell lung carcinoma	DSMZ	Roswell Park Memorial Institute medium (RPMI-1640)	Sigma-Aldrich (Steinheim, Germany)
**HeLa**	Human cervical adenocarcinoma	ATCC	DMEM High Glucose	Sigma-Aldrich (Steinheim, Germany)
**PC-3**	Human prostate adenocarcinoma	ATCC	DMEM High Glucose	Sigma-Aldrich (Steinheim, Germany)
**Sf9**	Pupal ovarian tissue of the fall army worm, *Spodoptera frugiperda*	Invitrogen Life Technologies	Ex-Cell™ 420	Sigma-Aldrich (Steinheim, Germany)
**SKOV-3**	Human ovarian adenocarcinoma	ATCC	McCoy's 5A	Sigma-Aldrich (Steinheim, Germany)

**St-T1b**	Human endometrial stroma, immortalized	University of Münster, Germany	DMEM/Ham's F12 medium, supplemented with insulin (1 μg/mL)	Sigma-Aldrich (Steinheim, Germany)
**T98-G**	Human glioblastoma	ATCC	Eagle's Minimum Essential Medium (EMEM) modified to contain Earle's Balanced Salt Solution, non-essential amino acids, 1 mM sodium pyruvate, and 1500 mg/L sodium bicarbonate	ATCC (Manassas, VA, USA)
**U-251 MG**	Human glioblastoma	ATCC	EMEM modified to contain Earle's Balanced Salt Solution, non-essential amino acids, 1 mM sodium pyruvate, and 1500 mg/L sodium bicarbonate	ATCC (Manassas, VA, USA)

[a] In case of mammalian cell lines, all growth media were supplemented with 10% FBS, 2 mM glutamine, 100 U/mL of penicillin, 100 μg/mL of streptomycin, and 0.25 μg/mL of amphotericin B.

Table S2. Suggested initial cell seeding densities (number of cells per well) for the resazurin-based viability assay in different cell lines.

**Cell line**	**Plate format**	**Time post-seeding**	**Detected optical characteristic**			
			Fluorescence (ex 540 nm, em 590 nm)		*Absorbance ratio (570 nm / 600 nm)*	
			Optimal # of cells per well [a]	*Z-factor* [b]	*Optimal # of cells per well* [a]	*Z-factor* [b]
**A-549**	96-well	72 h	780-1560	0.88	780-1560	0.73
	12-well	96 h	ND		10,000-20,000	0.90
**DU 145**	96-well	24 h	6300-12,500	0.88	6300-12,500	0.80
	96-well	48 h	3100-6300	0.90	6300-12,500	0.79
	96-well	72 h	1560-3100	0.89	2300-3125	0.75
	12-well	96 h	ND		5000-10,000	0.51
**HCC-44**	96-well	48 h	780-1560	0.85	1560-3100	0.75
	96-well	72 h	780-1560	0.81	780-1560	0.55
	12-well	96 h	ND		2500-5000	0.83
**HeLa**	96-well	48 h	3400-6800	0.78	14,000-27,000	0.75
	96-well	72 h	1300-2600	0.84	2600-5300	0.63
**PC-3**	96-well	24 h	13,000-25,000	0.82	13,000-25,000	0.89
	96-well	48 h	6300-12,500	0.69	6300-12,500	0.69
	96-well	72 h	3100-6300	0.79	3100-6300	0.59
	12-well	96 h	ND		10,000-20,000	0.63
**SKOV-3**	96-well	72 h	1300-2600	0.76	2600-5300	0.70
**St-T1b**	96-well	72 h	3300-6600	0.81	6600-13,000	0.69
**T98-G**	96-well	48 h	6300-12,500	0.62	6300-12,500	0.68
	96-well	72 h	3100-6300	0.64	6300-12,500	0.55
	96-well	96 h	1600-3100	0.64	3100-6300	0.50
	96-well	96 h (H, 37 °C)	2400-3100	0.44	2400-3100	0.36
	12-well	96 h	ND		5000-10000	0.55
**U-251 MG**	96-well	48 h	6300-12,500	0.78	13,000-25,000	0.73

96-well	72 h	3100-6300	0.58	3100-6300	0.53
96-well	96 h	1600-3100	0.63	1600-3100	0.75
96-well	96 h (H, 37 °C)	780-1600	0.46	780-1600	0.53
12-well	96 h	ND		10,000-20,000	0.54

[a] The optimization was conducted at 50 μM initial concentration of resazurin.
[b] The Z-factor was calculated based on the data measured the highest cell number (non-treated or vehicle-treated) within the indicated linear range following 60 min (fluorescence intensity) or 90 min (absorbance ratio) incubation with resazurin.
In red, Z-factor values of < 0.5 are shown. (H) indicates growing of cells under hypoxic conditions (1% of O_2 for 72 h) and ND stands for no data measured.

Table S3. Comparison of the technical characteristics of the resazurin assay and the 3-(4,5-dimethyl-thiazol-2-yl)-2,5-diphenyltetrazolium bromide (MTT) assay.

Characteristic	Resazurin assay	MTT assay
Generally advised concentration of the sensor	13 μg/mL (50 μM)	0.5 mg/mL (1.5 mM)
Readout format	Fluorescence or absorbance	Absorbance
Requires auxiliary reagents	No	Yes, formazan solubilization solution
Assay duration (including incubation with sensor)	0.5 h – 2 h	2 h – 8 h
Possibility of measurement during incubation with sensor	Yes	No
Major source of measurement uncertainty	Unequal seeding of cells to different wells	Unequal seeding of cells to different wells; Accidental removal of the formed formazan prior to solubilization
Consumption of laboratory plastic (pipette tips)	Medium (the tips need to be changed once after removal of growth medium, PBS wash, and addition of sensor)	High (the tips need to be changed once after addition of sensor and removal of medium + MTT, but for each well after addition of the solubilization solution)
Possibility to wash out the sensor and continue treatment of live cells	Yes	No
General flexibility	High (several parameters such as readout type or assay duration can be tested simultaneously)	Low (requires extensive optimization prior to the routine use)

The table was compiled based on our experimental data and the data available from the following literature sources ([1,2] references 15 and 38 in the main text):

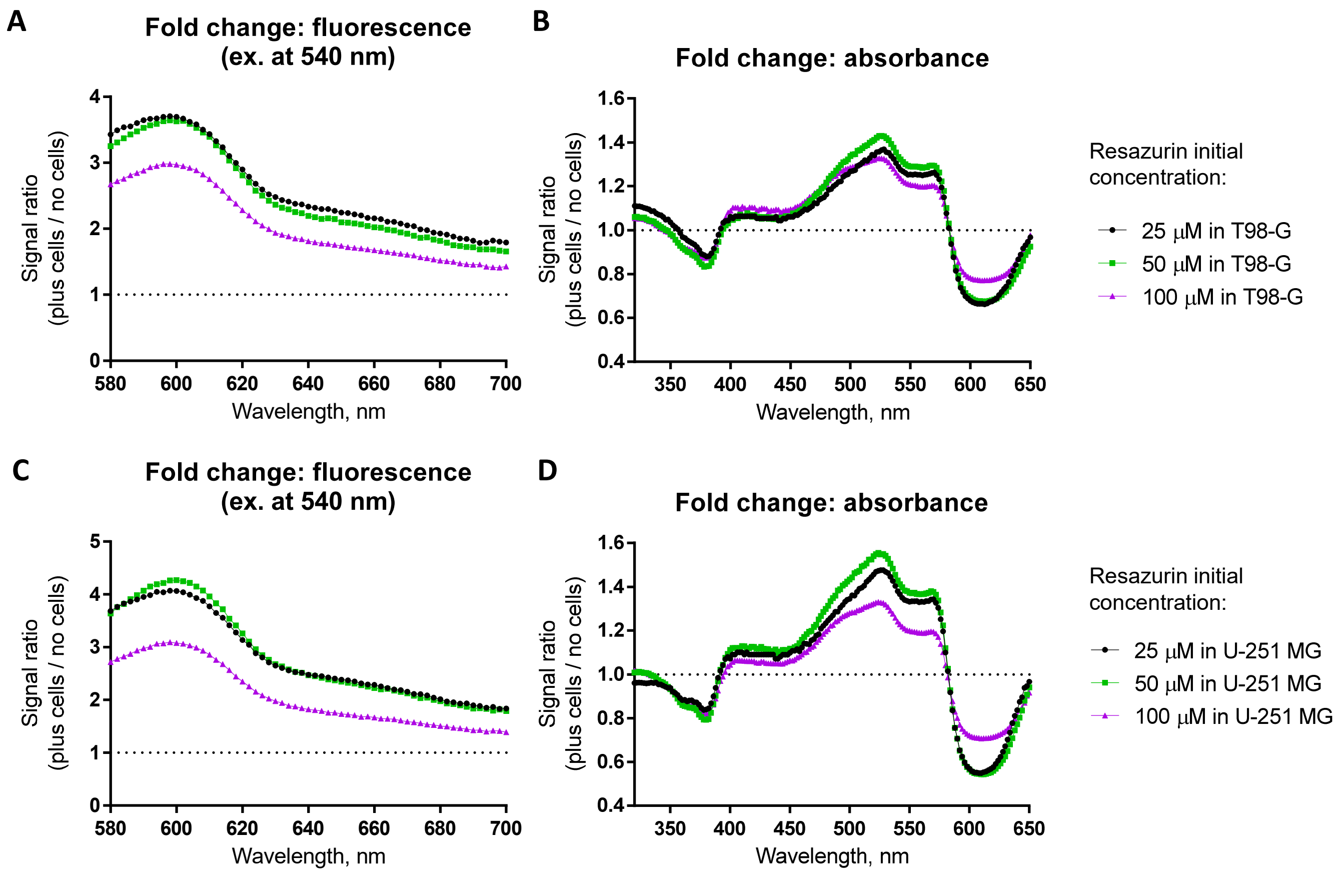

Figure S1. Fold change of resazurin spectra (A, C: fluorescence; B, D: absorbance) in the presence vs. absence of cells.

The spectra were measured after 2 h incubation of resazurin on the 96-well plate; in the case of incubation with cells, T98-G (A-B) or U-251 MG (C-D) cell lines were used (at 72 h post-seeding, initial seeding density of 3125 cells/well). The dotted line indicates no change (i.e., no dependence of the measured physical characteristic on the resazurin-to-resorufin transition in cells). A fold change above 1 indicates an increase in the measured signal during incubation with cells, and a fold change below 1 indicates a decrease in the measured signal during incubation with cells. The initial concentration of resazurin is indicated in the legend on the right; in each graph, an average of duplicates ± SEM from a single representative experiment is shown.

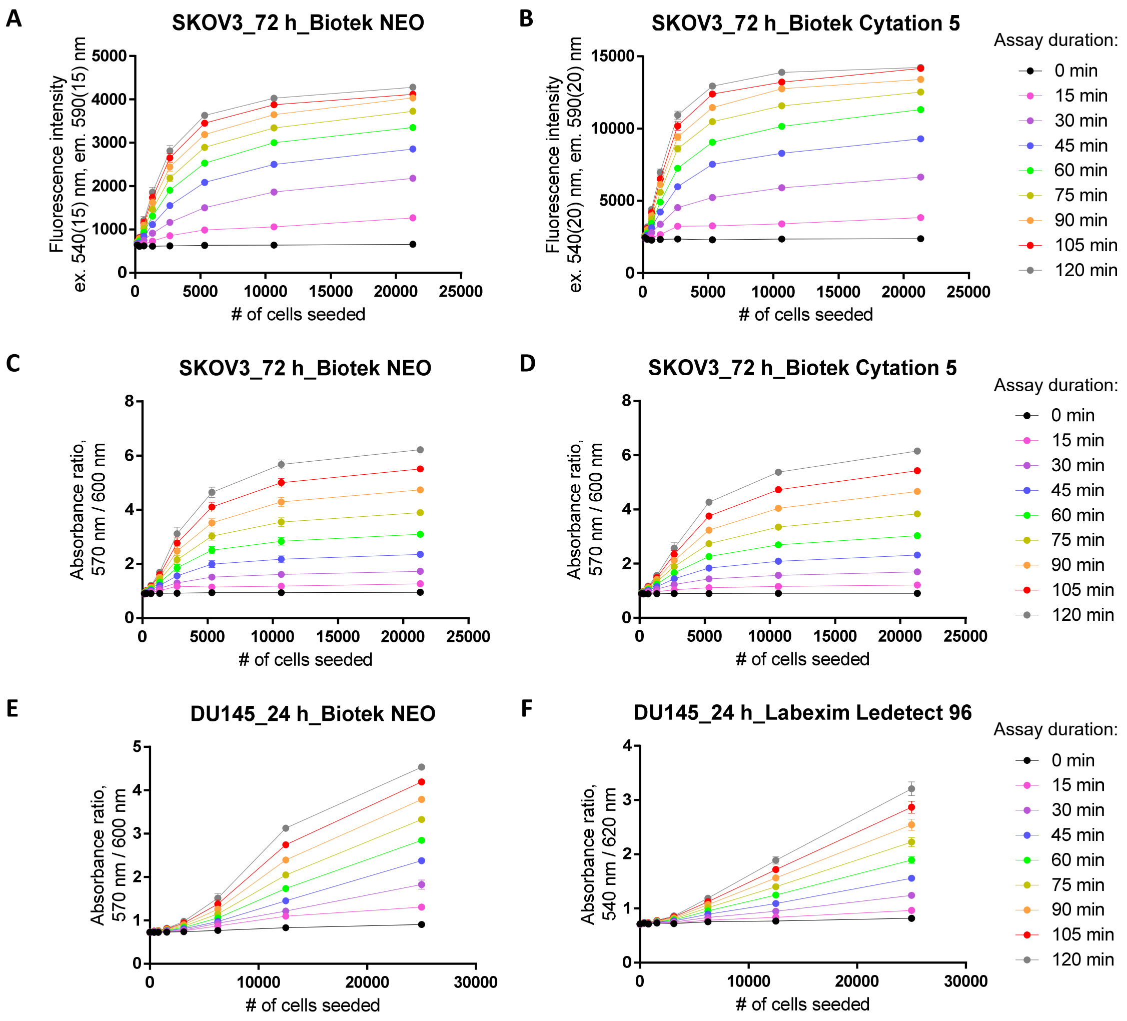

Figure S2. Example of absolute scale differences in detecting fluorescence (A, B) or absorbance ratio (C-F) during establishment of the dynamic range of resazurin assay using different apparatuses.

In A, C, and E, a Biotek NEO microplate reader was used (fluorescence: 15 nm slit width, absorbance measured at 570 nm and 600 nm); in B and D, a Biotek Cytation 5 microplate reader was used (fluorescence: 20 nm slit width, absorbance measured at 570 nm and 600 nm); in F, a Labexim Ledetect 96 was used (absorbance measured at 540 nm and 620 nm using light-emitting diodes as light source). Please note the differences of the y-scale values in A vs. B and in E vs. F. Measurements were carried out in SKOV-3 (A-D at 72 h post-seeding) or in DU 145 cells (E-F at 24 h post-seeding) at 50 µM resazurin (incubation times with resazurin indicated in the legend on the right). In each graph, an average of triplicates ± SEM from a single representative experiment is shown.

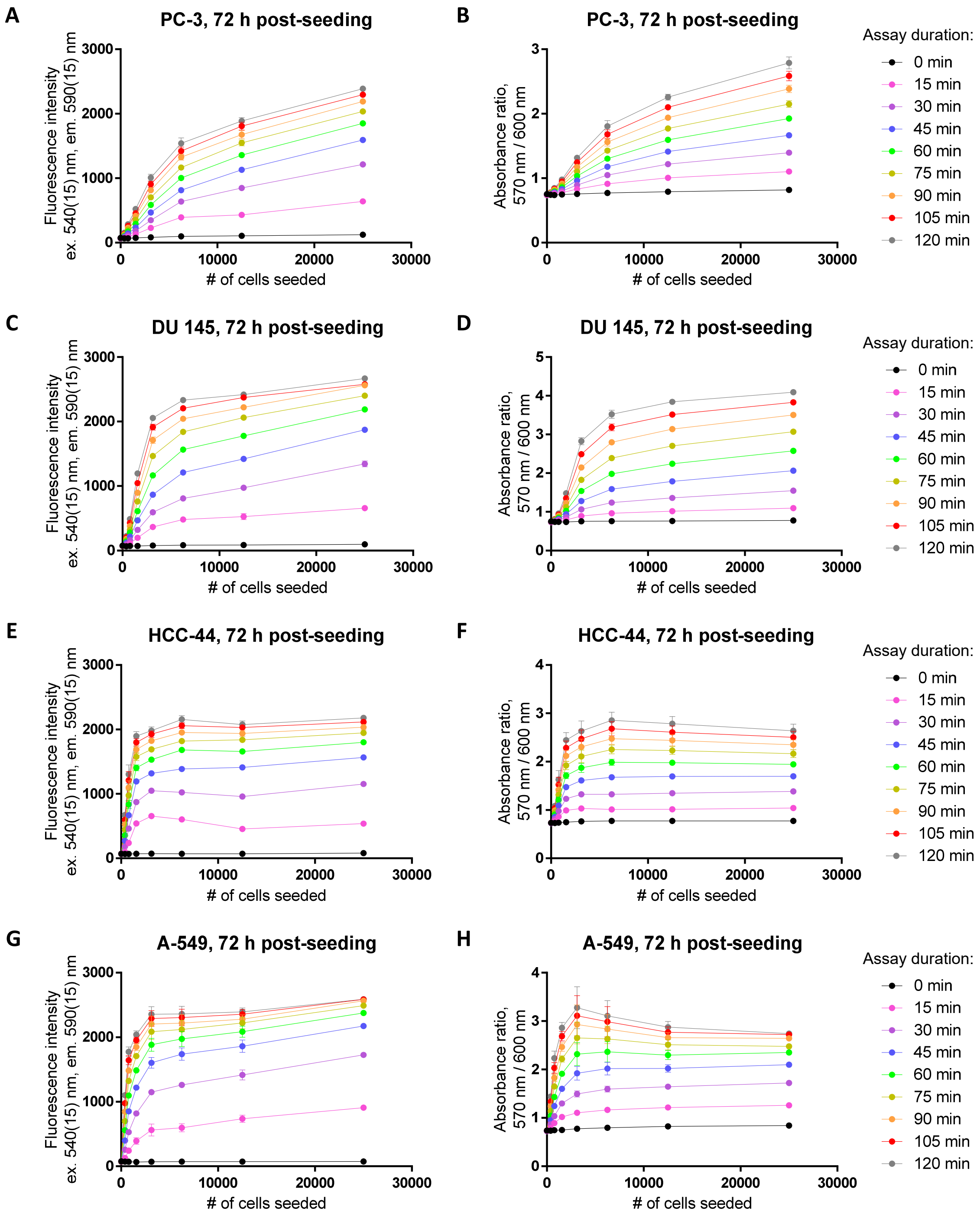

Figure S3. Establishment of the dynamic range of resazurin assay in the prostate cancer (A-D) or lung adenocarcinoma (E-H) cell lines at 72 h post-seeding.

Detected optical characteristic: fluorescence intensity (excitation at 540 nm) in A, C, E, and G; and an absorbance ratio (570 nm / 600 nm) in B, D, F, and H. Measurements were carried out at 50 µM resazurin (incubation times with resazurin indicated in the legend on the right). In each graph, the average of triplicates ± SEM from a single representative experiment is shown.

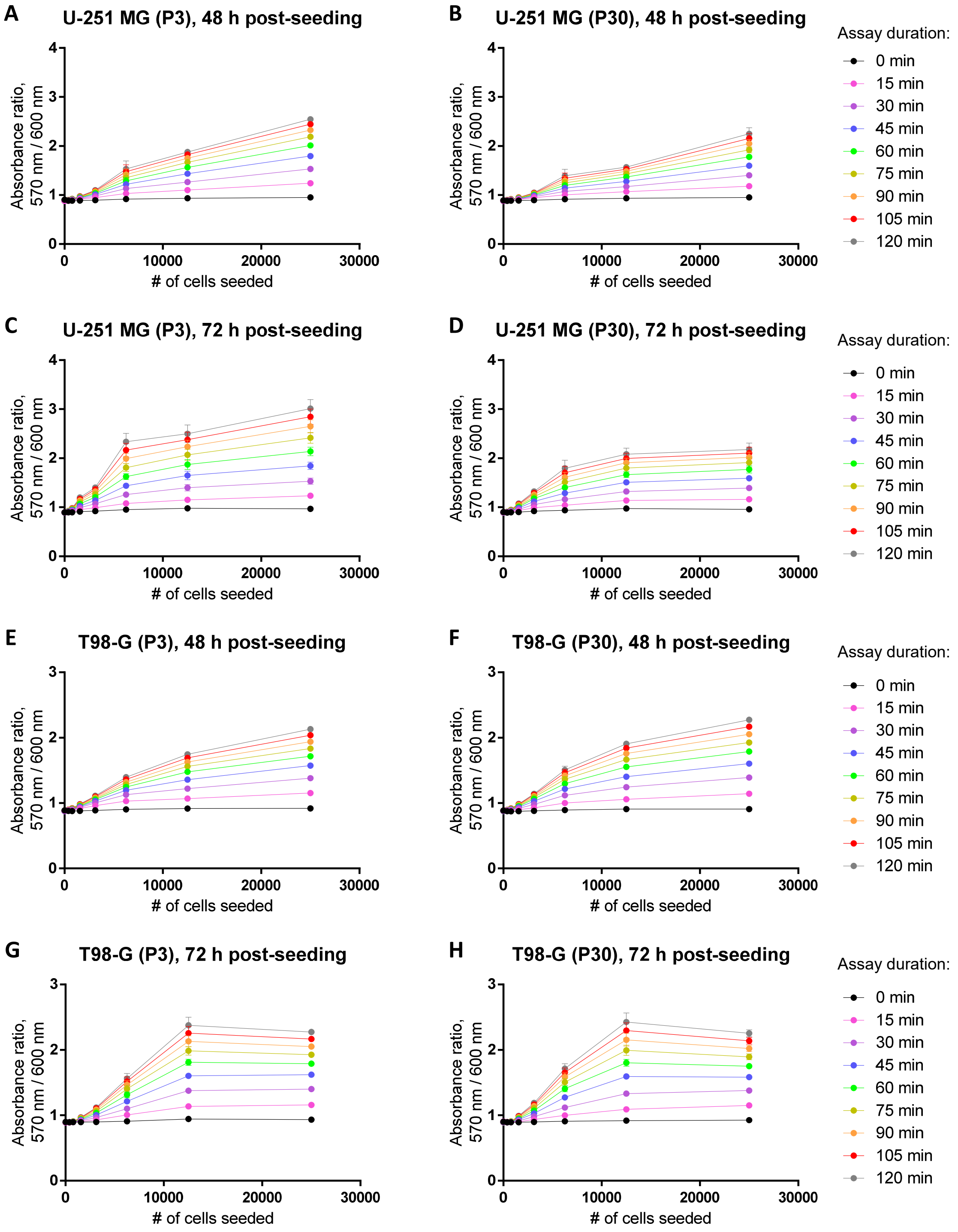

Figure S4. Establishment of the dynamic range in the resazurin assay for different passages (P3: A, C, E, and G; P30: B, D, F, and H) of glioblastoma cell lines U-251 MG (A-D) or T98-G (E-H) at 48 h (A-B, E-F) or at 72 h (C-D, G-H) post-seeding.

Detected optical characteristic: absorbance ratio (570 nm / 600 nm). All experiments were carried out in the 96-well plate format at 50 µM resazurin (incubation times with

resazurin indicated in the legend on the right). In each graph, the average of triplicates ± SEM from a single representative experiment is shown.

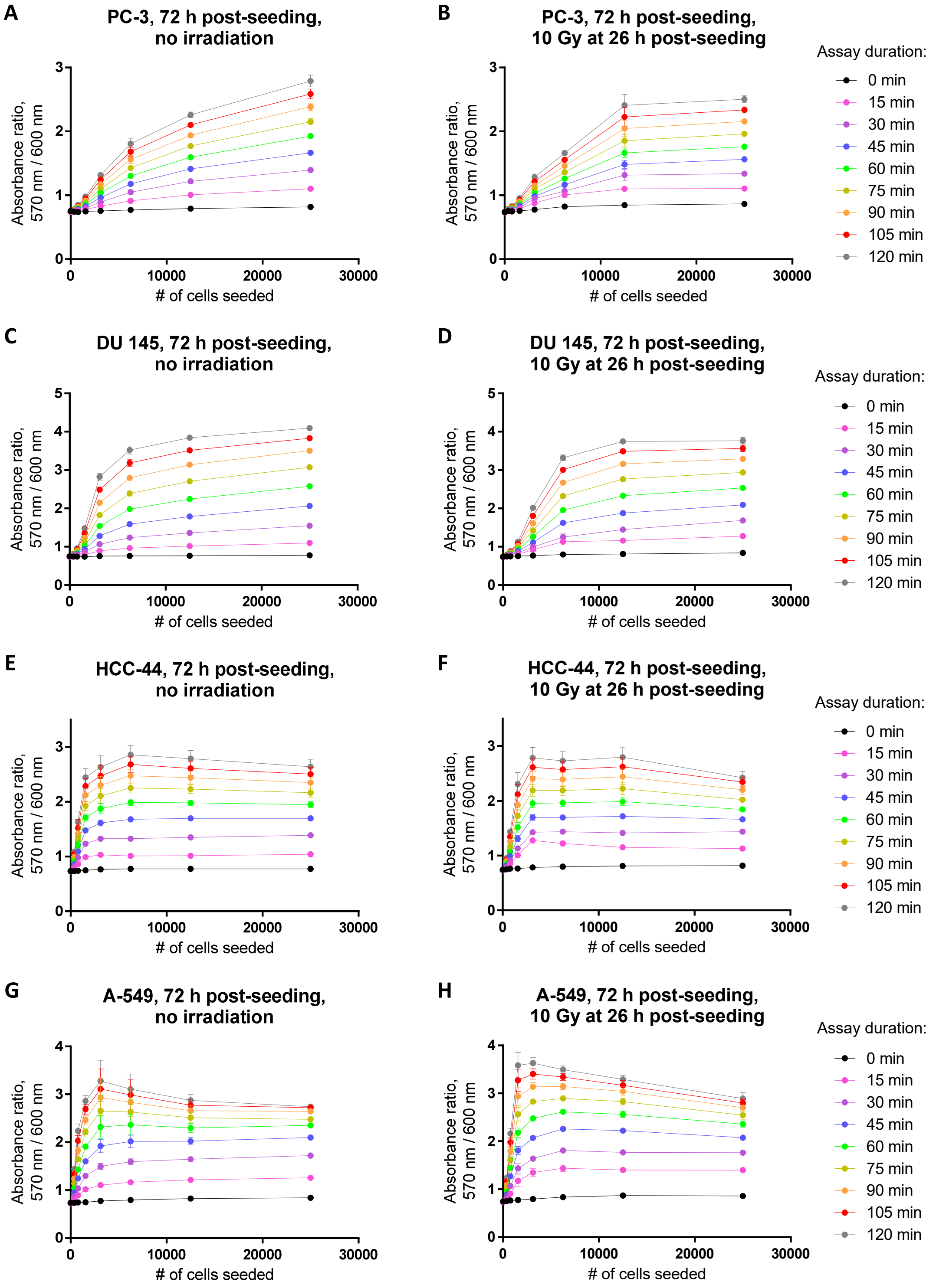

Figure S5. Establishment of the dynamic range in the resazurin assay for prostate cancer (A-D) or lung adenocarcinoma (E-H) cell lines following irradiation at 10 Gy (96-well format).

Irradiation of cells (B, D, F, H) was carried out 26 h post-seeding and measurement of viability was performed at 72 h post-seeding. Non-irradiated plates (A, C, E, G) were also transported to the radiation facility but kept in the control room. Detected optical characteristics: absorbance ratio (570 nm / 600 nm); measurements were carried out at 50 µM resazurin (incubation times with resazurin indicated in the legend on the right). In each graph, an average of triplicates ± SEM from a single representative experiment is shown.

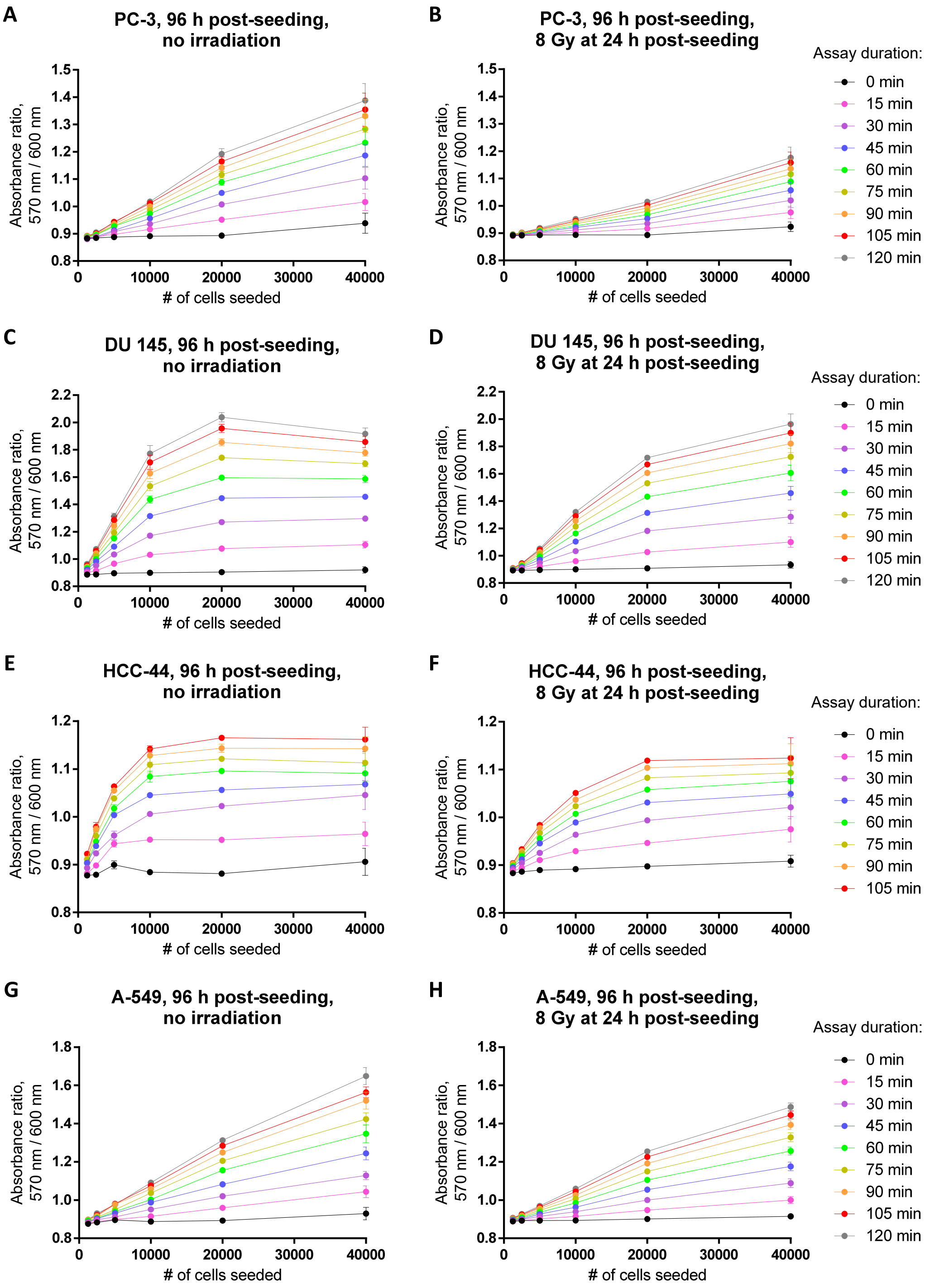

Figure S6. Establishment of the dynamic range in the resazurin assay for prostate cancer (A-D) or lung adenocarcinoma (E-H) cell lines following irradiation at 8 Gy (12-well format).

Irradiation of cells (B, D, F, H) was carried out at 24 h post-seeding and measurement of viability was performed at 96 h post-seeding. Non-irradiated plates (A, C, E, G) were also transported to the radiation facility but kept in the control room. Detected optical characteristics: absorbance ratio (570 nm / 600 nm); measurements were carried out at 50 μM resazurin (incubation times with resazurin indicated in the legend on the right). In each graph, the average of triplicates ± SEM from a single representative experiment is shown.

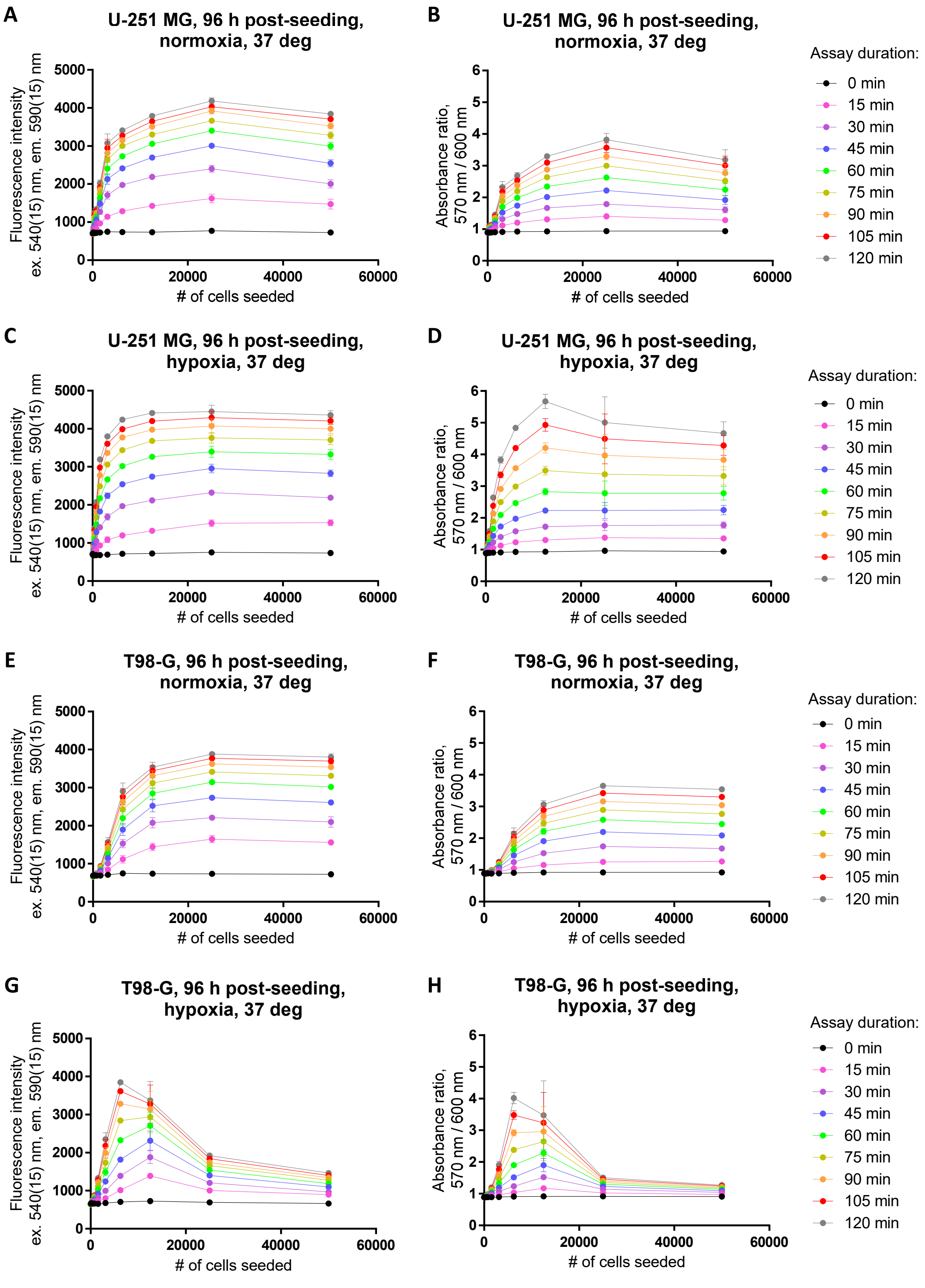

Figure S7. Establishment of the dynamic range in the resazurin assay following normoxic (A-B, E-F) vs. hypoxic treatment (C-D, G-H) at 37 °C for glioblastoma cell lines (A-D, U-251 MG; E-H, T98-G).

For hypoxic treatment, cells were kept in a hypoxic chamber (1% O_2) for 72 h prior to measurement of viability (at 96 h post-seeding). Detected optical characteristics:

fluorescence intensity (excitation at 540 nm) in A, C, E, and G; absorbance ratio (570 nm / 600 nm) in B, D, F, and H. Measurements were carried out at 50 µM resazurin (incubation times with resazurin indicated in the legend on the right). In each graph, the average of duplicates or triplicates ± SEM from a single representative experiment is shown.

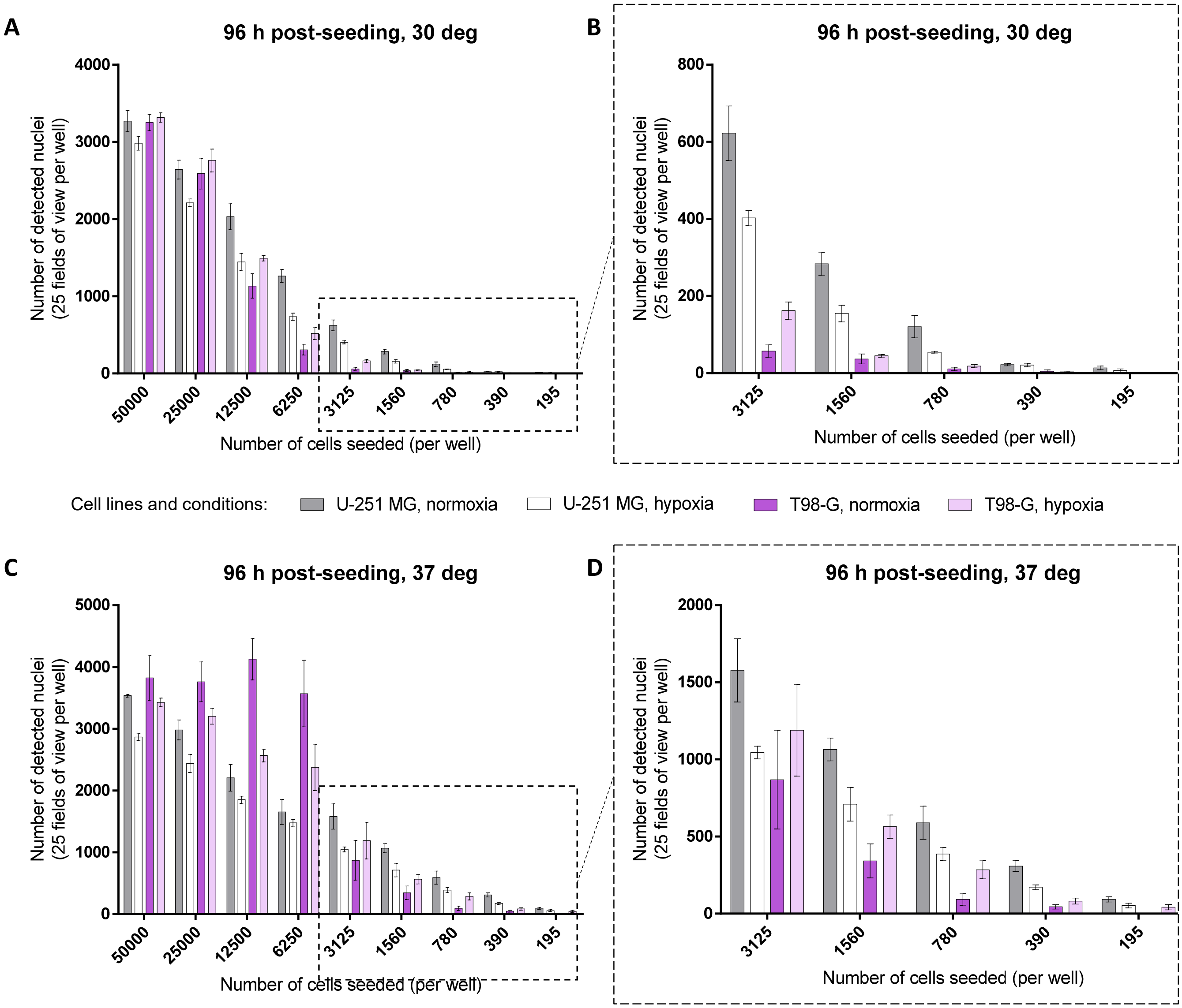

Figure S8. Detection of the number of nuclei using microscopy assay following normoxic vs. hypoxic treatment of glioblastoma cell lines (indicated under the graphs) at different seeding densities and temperatures.

For hypoxic treatment, cells were kept in a hypoxic chamber (1% O_2) for 72 h prior to measuring the viability (at 96 h post-seeding). The nuclei were counted using automatic detection algorithm based on the DAPI staining. (A) Hypoxic or normoxic treatment carried out at 30 °C; seeding density of 195-50,000 cells per well; (B) same data as in A at low seeding density numbers, Y-axis extent adjusted accordingly. (C) Hypoxic or normoxic treatment carried out at 37 °C; seeding density of 195-50,000 cells per well; (D) same data as in C at low seeding density numbers, Y-axis extent adjusted accordingly. Average of triplicates ± SEM from a single representative experiment is shown.

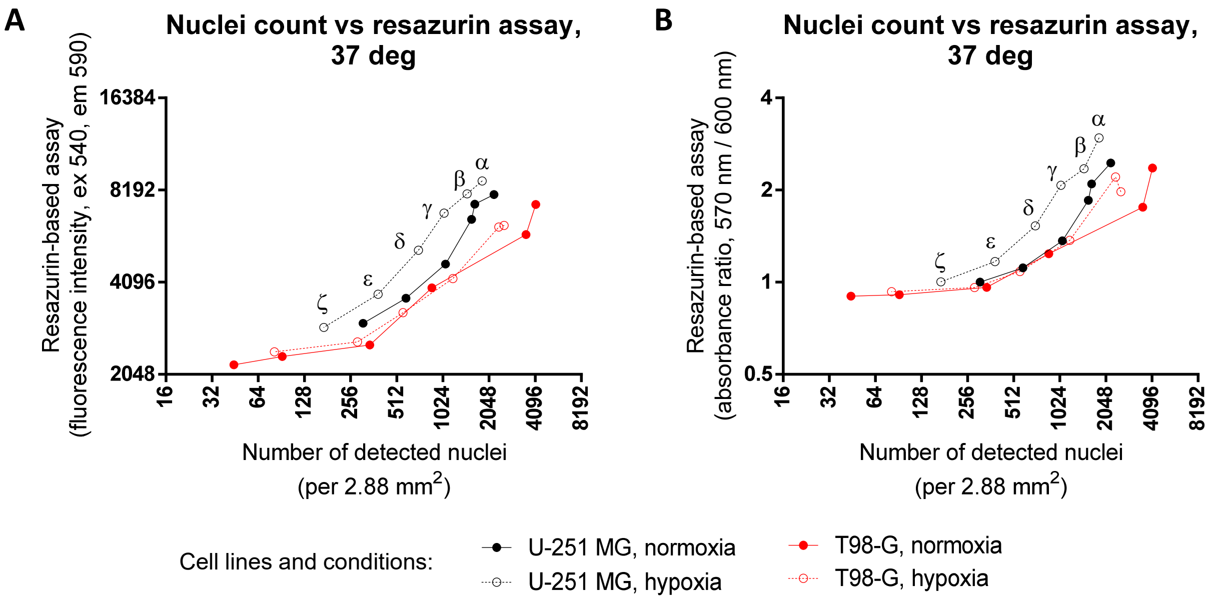

Figure S9. Comparison of data reflecting cellular viability measured using detection of metabolic activity and detection of the number of nuclei following normoxic vs. hypoxic treatment of glioblastoma cell lines at 37 °C.

Y-scale, resazurin assay; X-scale, microscopy assay; cell line names indicated under the graphs. Different initial seeding densities were used (for better visualization, indicated for the left-most curve only: α, 12,500 cells per well; β, 6250 cell per well; γ, 3130 cell per well; δ, 1560 cells per well; ε, 780 cells per well; ζ, 390 cells per well). For hypoxic treatment, cells were kept in a hypoxic chamber (1% O_2) for 72 h prior to measuring the viability (at 96 h post-seeding). In the resazurin assay, measurements were carried out at 50 µM resazurin; fluorescence intensity (excitation at 540 nm) was detected in A (after 45 min incubation with resazurin) and absorbance ratio (570 nm / 600 nm) in B (after 60 min incubation with resazurin). The nuclei were counted in 25 fields of view (dimensions of one field of view: 292 µm × 395 µm) using an automatic detection algorithm based on the DAPI staining. Please note that the axes are logarithmic. In each graph, an average of triplicates from a single representative resazurin and a single representative microscopy experiment is shown; for better clarity, vertical and horizontal error bars are omitted.

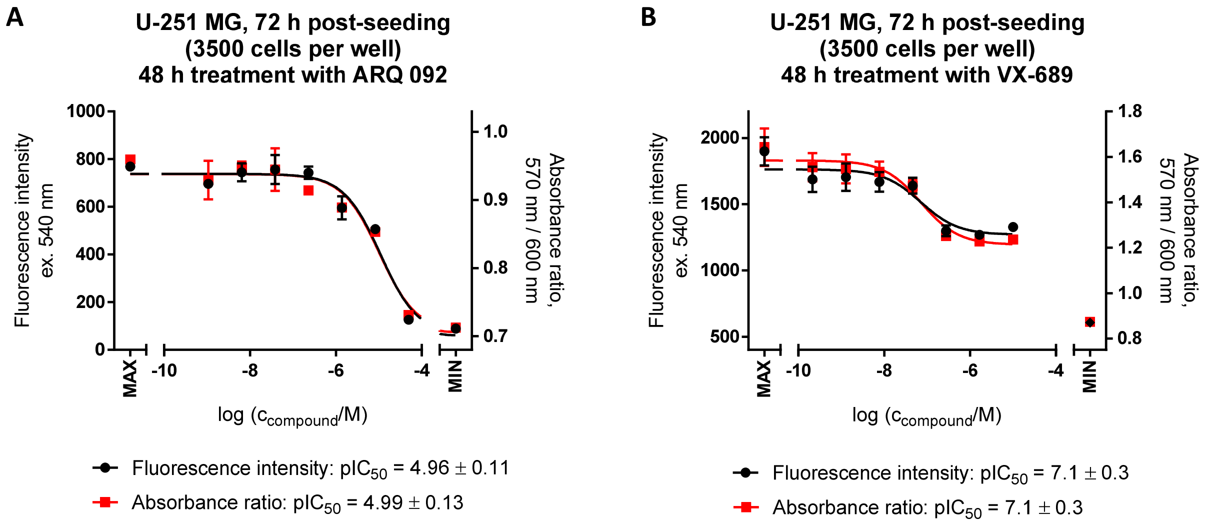

Figure S10. Comparison of the dose–response curves measured using different readouts for a cytotoxic (A) or a cytostatic (B) compound.

U-251 MG cells were treated with compounds for 48 h prior to measuring the viability (at 72 h post-seeding). Measurements were carried out at 50 µM resazurin. An incubation time of 60 min was used with resazurin to measure the fluorescence intensity (excitation at 540 nm), and 75 min was used to measure the absorbance ratio (570 nm / 600 nm). The readouts are indicated on different Y-axes (left: fluorescence intensity, right: absorbance ratio). Graph A shows average of duplicates ± SEM from a single representative experiment and graph B shows pooled data from two independent experiments. The following controls were used: MAX = non-treated cells, MIN = well without cells. The curves were fitted to the three-parameter logarithmic equations (a MIN datapoint was included in the analysis for A but excluded from analysis for B). Below the graphs, negative logarithms of the IC_{50} values ± SEM calculated for each readout type are presented.

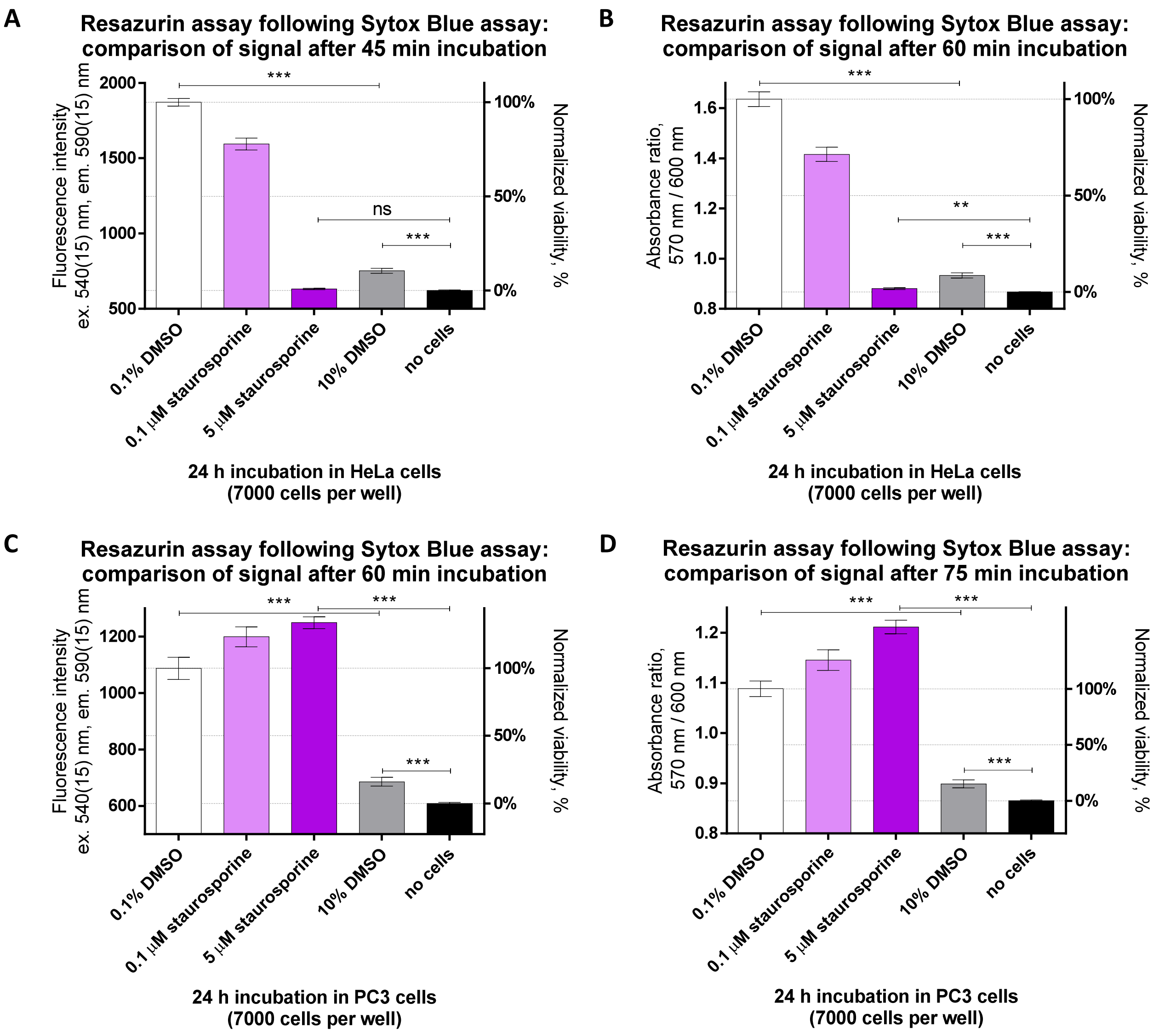

Figure S11. Resazurin assay in HeLa or PC3 cell lines: comparison of different negative controls.

Cells were treated with compounds (conditions indicated below graphs) for 24 h prior to measurement of viability (at 48 h post-seeding). Values of fluorescence intensity (A, C) or absorbance ratio (B, D) are shown following incubation of cells with 50 µM resazurin (the incubation time corresponds to the end of linear range; details shown in graph headings). In each graph, an average of six or more technical replicates ± SEM from three independent experiments is shown. The statistical significance of pairwise comparisons between the indicated columns was established using the unpaired two-tailed t-test (95% confidence level) with Welch's correction: *** indicates $P \leq 0.001$, ** indicates $P \leq 0.01$, ns stands for not significant.

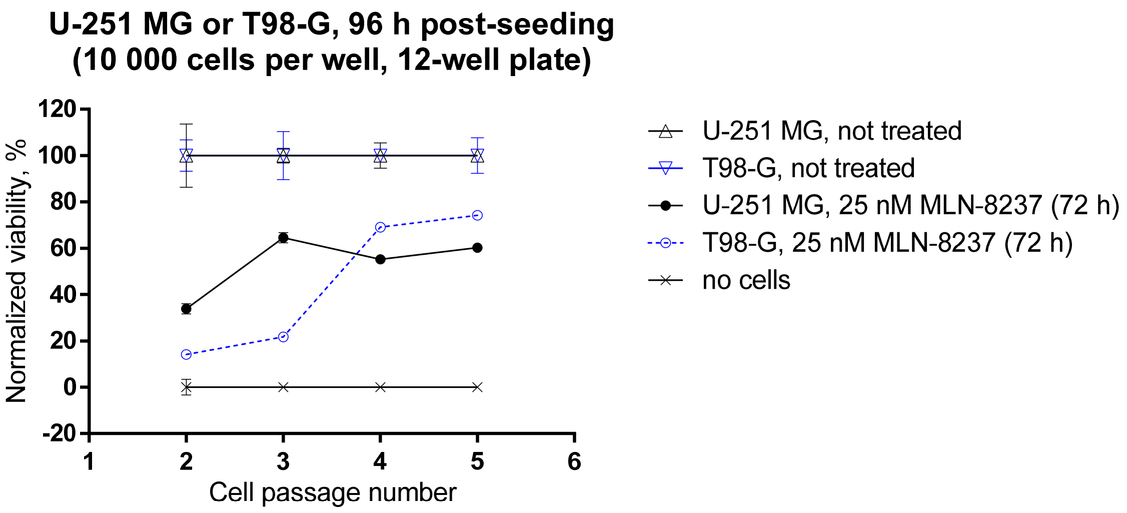

Figure S12. Effect of Aurora A inhibitor MLN-8237 (25 nM) on viability of glioblastoma cell lines dependent on the passage number of the latter.

Cell lines and treatment conditions are listed on the right. Detected optical characteristic: absorbance ratio (570 nm / 600 nm); measurements were carried out at 50 µM resazurin. The graph shows pooled data (mean normalized viability ± SEM) of a total of four independent experiments for each cell line. The normalization was performed using wells with non-treated cells (100% viability) and wells without cells (0% viability).

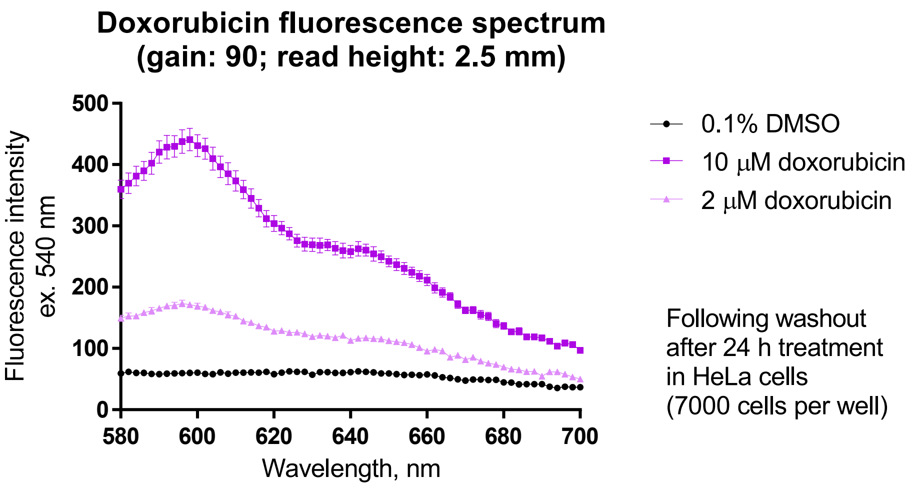

Figure S13. Fluorescence spectrum of HeLa cells treated with doxorubicin.

HeLa cells were seeded onto 96-well plate and, after 24 h, doxorubicin (final concentration: 10 µM or 2 µM) was treated in the usual cell culture medium (duration: 24 h). Prior to the measurement of doxorubicin fluorescence intensity, the cell culture medium was removed and PBS (supplemented with Ca^{2+} and Mg^{2+}) was added. The read height was adjusted to focus onto the bottom of the well. Pooled data (average ± SEM) from three independent experiments are shown.

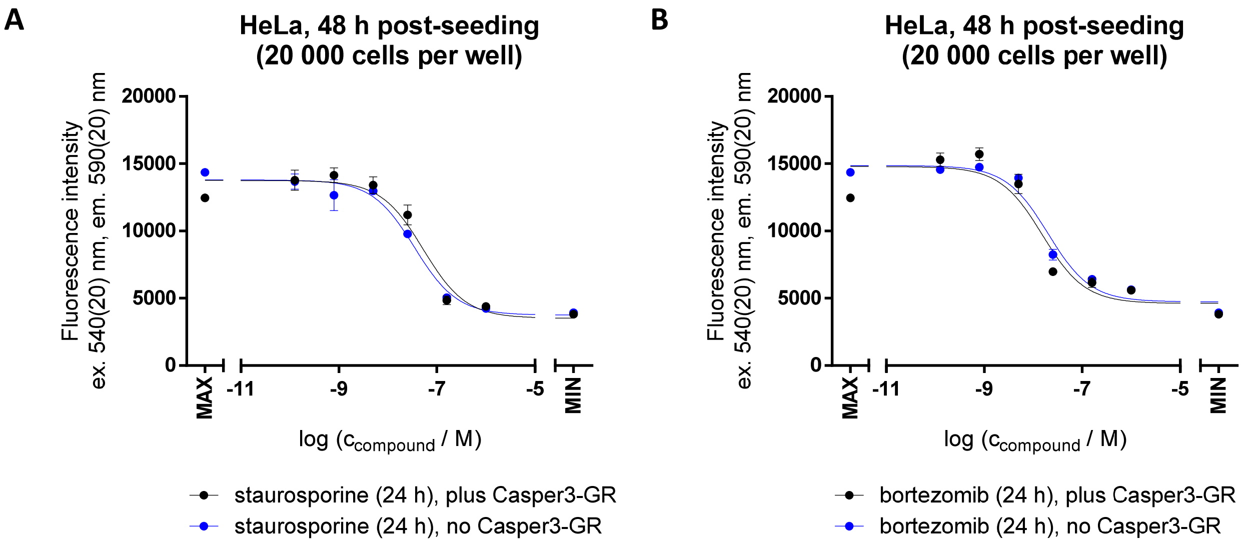

Figure S14. The results of resazurin assay are independent on the transfection of cells with Casper3-GR biosensor.

Non-transfected HeLa cells or HeLa cells transfected with Casper3-GR were seeded onto 96-well plates; after 24 h, variable doses of staurosporine (A) or bortezomib (B) were treated, accompanied with imaging of plates during the apoptosis assay. The apoptosis assay lasted for 23–24 h prior to measuring the viability (at 47–48 h post-seeding). Detected optical characteristics: fluorescence intensity (excitation at 540 nm); measurements were carried out at 50 µM resazurin (incubation time with resazurin: 90 min). In each graph, the average of duplicates ± SEM from a single representative experiment is shown; MAX corresponds to the cells treated with 0.1% DMSO and MIN corresponds to the cells treated with 10% DMSO.

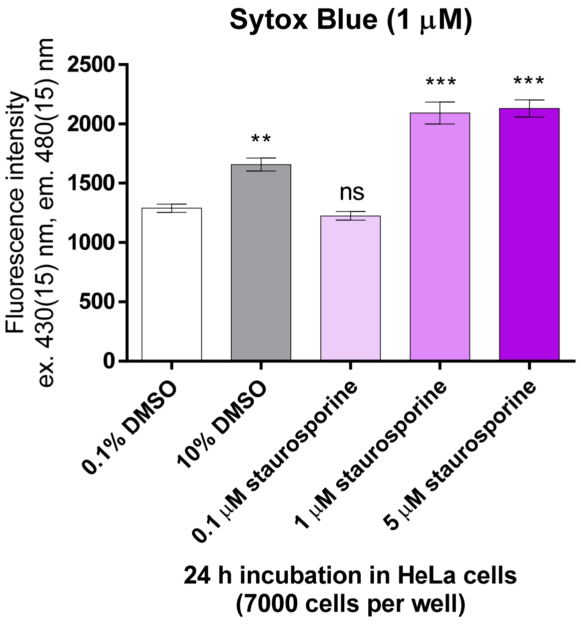

Figure S15. Sytox Blue assay results in HeLa cells following 24 h treatment with staurosporine or DMSO.

HeLa cells were seeded onto 96-well plate and, after 24 h, were treated with staurosporine (final concentration: 5 μM, 1 μM, or 0.1 μM) or DMSO (final percentage by volume: 10% or 0.1%) in the usual cell culture medium (duration: 24 h). Prior to the measurement of Sytox Blue fluorescence intensity, the cell culture medium was removed and 1 μM solution of Sytox Blue in PBS (supplemented with Ca^{2+} and Mg^{2+}) was added. Following 10 min incubation, the fluorescence intensity (excitation at 430 nm) was measured using the area scan mode (5×5 fields of view per well). The read height was adjusted to focus onto the bottom of the well. Pooled data (mean intensity per well ± SEM) from four or more technical replicates across six independent experiments are shown. The statistical significance of difference between the cells treated with 0.1% DMSO (negative control, no necrosis/late apoptosis) vs. other solutions was assessed using one-way ANOVA with Dunnett's test for multiple comparisons; *** indicates $P \leq 0.001$, * indicates $P \leq 0.05$.

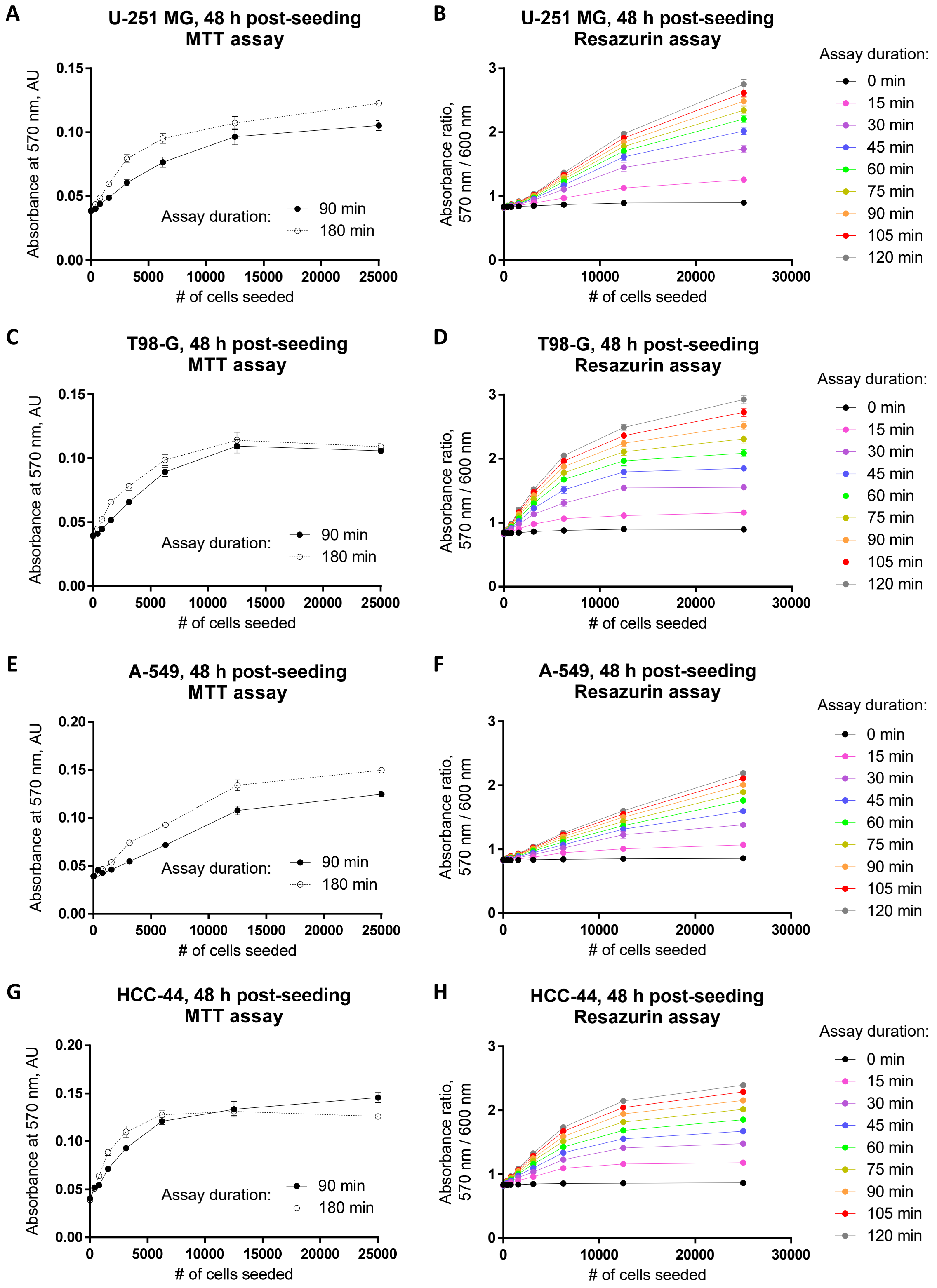

Figure S16. Comparison of the dynamic range in the resazurin assay and the MTT assay.

U-251 MG (A-B), T98-G (C-D), A-549 (E-F,) or HCC-44 (G-H) cells were seeded at different densities onto the 96-well plates and, after 48 h, the MTT assay (A, C, E, G) or resazurin assay (B, D, F, H) was carried out. The incubation time with MTT (0.5 mg/mL) was varied (indicated in the legends at the bottom of each graph). The solubilization of the formed formazan prior to measuring the absorbance (570 nm) was carried out using 4 h treatment of wells with 10% sodium dodecyl sulphate in 0.01 M hydrochloric acid. In case of the resazurin assay, a 50 µM sensor was used; absorbance ratio (570 nm / 600 nm) was measured at the indicated timepoints (legends on the right) during the incubation of cells with the sensor. In each graph, the average of triplicates ± SEM pooled from two independent experiments is shown.

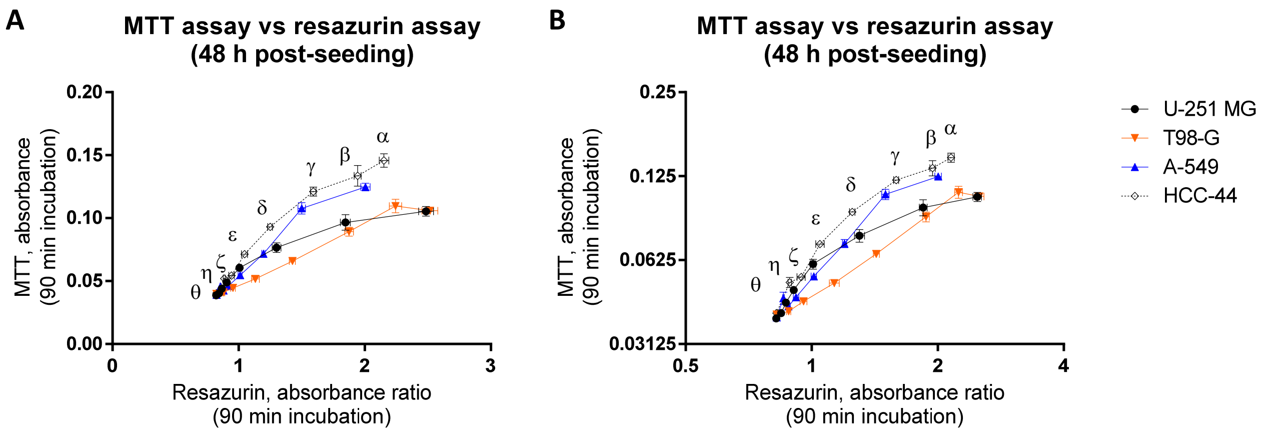

Figure S17. Correlation between the data measured using the resazurin assay and MTT assay.

Y-scale, MTT assay; X-scale, resazurin assay; the scales are linear in (A) and logarithmic in (B). Cell line names are indicated in the legend on the right. Different initial seeding densities were used (for better visualization, indicated for the left-most curve only: α, 25,000 cells per well; β, 12,500 cell per well; γ, 6250 cell per well; δ, 3130 cells per well; ε, 1560 cells per well; ζ, 380 cells per well; η, 190 cells per well; θ, 0 cells). In the resazurin assay, measurements were carried out at 50 µM resazurin; absorbance ratio (570 nm / 600 nm) following the 90 min incubation with resazurin is depicted. In the MTT assay, measurements were carried out at 0.5 mg/mL of MTT; the data for the 90 min incubation is depicted. The solubilization of the formed formazan prior to measurement of absorbance (570 nm) was carried out using 4 h treatment of wells with 10% sodium dodecyl sulphate in 0.01 M hydrochloric acid. In each graph, the average of triplicates ± SEM pooled from two independent experiments is shown.

Protocol S1. Establishment of the dynamic range of resazurin assay.

The vendors for the reagents used can be found under Section 2.1 of the main text.

Part A. Seeding of cells

- Autoclave the distilled water and cool down to room temperature
    - Volume needed per one measurement plate: *ca* 5 mL
- Pre-warm the sterile phosphate buffered saline (PBS), sterile trypsin, and sterile cell growth medium to 37 °C or room temperature
    - Volume of medium needed per one measurement plate: *ca* 7 mL in case if 100 µL volume per well is used

- Place under the laminar pipetting vessels, centrifuge tubes for collection and dilutions of cell suspension, set of pipettes and suitable pipette tips, and one or more 96-well tissue culture-treated plates with flat bottom
  - It is possible to test two cell lines on one plate; for different incubation times post-seeding, different plates should be used
- Trypsinize the cells according to the usual cell culture protocol, collect the cell suspension, and count the cells using the cell counter or other routine methodology
- Using the pipetting vessel and a multichannel pipette, transfer 100 µL of growth medium per well into the columns 3-11 (rows B-G) of the measurement plate
  - The rows A and H and the columns 1 and 12 need to be kept empty; at the end of the seeding, autoclaved water (a' 100 µL per well) needs to be added to each empty well to reduce the measurement uncertainty related to evaporation of liquid
  - For prolonged incubation times (over 48 h), the volume of medium can be increased (up to 200 µL); in this case, use correspondingly larger volumes in all the subsequent steps
- Perform the necessary calculations and prepare a diluted cell suspension containing 200 000 of cells per 1 mL of suspension (equals 40 000 cells per 200 µL)
  - The required volume of suspension is *ca* 2 mL per one measurement plate if one cell line is used
  - Transfer 200 µL of the diluted suspension to the wells in column 2 (rows B-G)

An example layout at this stage in case if two cell lines are used.

		1	2	3	4	5	6	7	8	9	10	11	12
	A												
Cell line 1	B		40 000 cells in 200 µL of medium 1	100 µL medium 1	100 µL medium 1	100 µL medium 1	100 µL medium 1	100 µL medium 1	100 µL medium 1	100 µL medium 1	100 µL medium 1	100 µL medium 1	
Cell line 1	C		40 000 cells in 200 µL of medium 1	100 µL medium 1	100 µL medium 1	100 µL medium 1	100 µL medium 1	100 µL medium 1	100 µL medium 1	100 µL medium 1	100 µL medium 1	100 µL medium 1	
Cell line 1	D		40 000 cells in 200 µL of medium 1	100 µL medium 1	100 µL medium 1	100 µL medium 1	100 µL medium 1	100 µL medium 1	100 µL medium 1	100 µL medium 1	100 µL medium 1	100 µL medium 1	
Cell line 2	E		40 000 cells in 200 µL of medium 2	100 µL medium 2	100 µL medium 2	100 µL medium 2	100 µL medium 2	100 µL medium 2	100 µL medium 2	100 µL medium 2	100 µL medium 2	100 µL medium 2	
Cell line 2	F		40 000 cells in 200 µL of medium 2	100 µL medium 2	100 µL medium 2	100 µL medium 2	100 µL medium 2	100 µL medium 2	100 µL medium 2	100 µL medium 2	100 µL medium 2	100 µL medium 2	
Cell line 2	G		40 000 cells in 200 µL of medium 2	100 µL medium 2	100 µL medium 2	100 µL medium 2	100 µL medium 2	100 µL medium 2	100 µL medium 2	100 µL medium 2	100 µL medium 2	100 µL medium 2	
	H												

- Using the multichannel pipette, transfer 100 µL of the cell suspension from column 2 to column 3, mix at least 8 times and transfer 100 µL of the obtained suspension to the next column, and ensure continue dilutions until column 10

    - After mixing the obtained suspension in column 10, discard 100 µL of the well contents so that all filled wells on the plate contain 100 µL of cell suspension (columns 2-10) or pure medium (control, column 11)
- Place the plate into 37 °C CO_2 incubator and incubate under the usual cell growth conditions for the planned amount of time (48–96 h)
    - For instance, if the dynamic range measurement is carried out for the assessment of the optimal cell number to be used in 48 h cytotoxicity experiment, then in the dynamic range optimization experiment, 72 h incubation post-seeding is required (because the cytotoxic compounds are applied at 24 h post-seeding—please see also Supplementary Protocol 2)

Part B. 48 h post-seeding or later: resazurin assay

- Pre-warm PBS supplemented with Ca^{2+} and Mg^{2+} to 37 °C or room temperature, switch on the microtiter plate reader, and prepare 2 pipetting vessels and a waste dish
    - If the machine enables the internal temperature control, set the incubator to 37 °C
- Prepare the solution of resazurin in PBS into a centrifuge tube (50 µM)
    - Volume required: 8 mL per one measurement plate
    - In case of small-scale measurements, preparation of one or several more concentrated stock solutions (e.g., 100 mM and/or 5 mM resazurin in distilled water) might be advisable, from which the necessary dilution into PBS can be subsequently made
        - The stock solutions can be stored at -20 °C for a couple of months
- Define the measurement protocol
    - If the machine enables the kinetic measurement mode: perform measurement for 2 h with the reading(s) taken after each 15 min
    - If the machine enables measurement of both fluorescence intensity and absorbance: measure both physical quantities
        - Fluorescence intensity: excitation at 540 nm or similar, emission at 590 nm or similar
        - Absorbance: 540-570 nm wavelength is suitable for monitoring the formation of resorufin within the course of the reaction; a 600-620 nm wavelength is suitable for monitoring consumption of resazurin within the course of the reaction
- The following work does not have to be carried out under the aseptic conditions unless the experiment presupposes continuation of the live cell treatment following the resazurin assay and washout of the sensor
- Using the multichannel pipette, remove the cell culture medium from the plate with cells
- Using the multichannel pipette and the pipetting vessel, wash the cells briefly with PBS supplemented with Ca^{2+} and Mg^{2+} (100 µL per well) to remove the trances of growth medium
    - This step is not necessary if the phenol red-free medium was used post-seeding
- Using the multichannel pipette, discard the PBS washing solution and transfer the resazurin solution from the pipetting vessel onto the cells (100 µL per well)
- Place the plate into the reader and start the measurement immediately

    - If the microplate reader settings can keep the plate closed with a lid, use the corresponding option; however, make sure that no condensate resulting from the temperature differences was formed on lid during the previous steps as the condensate may interfere with the readout signal
- For data analysis, pool the replicates and plot the XY graph picturing dependence of the readout on the number of cells seeded
    - According to the given protocol, the number of seeded cells starts at 20 000 cells per well (column 2) and is reduced 2-fold in each subsequent column; column 11 is the blank control
    - For multiple readouts, make one XY graph for each type of readout
    - If the kinetic measurement mode was used, plot the datapoints measured at different incubation times with resazurin onto the same graph as different series
    - From the XY graphs, determine the number of seeded cells that is within the linear range of the assay (if you plan to use multiple readouts later on, the number of seeded cells must be within the linear range for both readout formats)

Protocol S2. Application of the resazurin assay for assessment of the cytotoxic properties of the compounds of interest
The vendors for the reagents used can be found under Section 2.1 of the main text.
Part A. Seeding of cells

- Autoclave the distilled water and cool down to room temperature
    - Volume needed per one measurement plate: *ca* 3 mL
- Pre-warm the sterile phosphate buffered saline (PBS), sterile trypsin, and sterile cell growth medium to 37 °C or room temperature
    - Volume of medium needed per one measurement plate: *ca* 9 mL in case if 100 µL volume per well is used
- Place under the laminar pipetting vessels, centrifuge tubes for collection and dilutions of cell suspension, a set of pipettes and suitable pipette tips, and one or more 96-well tissue culture-treated plates with flat bottom
    - It is possible to test two cell lines or two seeding densities of a single cell line on one plate; for different incubation times post-seeding, different plates should be used
- Trypsinize the cells according to the usual cell culture protocol, collect the cell suspension, and count the cells using the cell counter or other routine methodology
- Perform the necessary calculations and prepare a diluted cell suspension according to the results of the dynamic range experiment (as a rule of thumb, for a 48 h experiment, the number of cells per well should be in the range of 2000-8000, whereas for a 72 h experiment, the number of cells per well should be in the range of 1500-5000)
    - The required volume of suspension is *ca* 9 mL per one measurement plate in case if 100 µL volume per well and a single cell line is used
    - Using the multichannel pipette and a pipetting vessel, transfer 100 µL of the diluted suspension to the wells in columns 1-12 (rows B-G)
    - The rows A and H need to be kept empty; at the end of the seeding, autoclaved water (100 µL per well) needs to be added to each empty well to reduce the measurement uncertainty related to evaporation of liquid
- Place the plate into 37 °C CO_2 incubator and incubate under the usual cell growth conditions for 24 h

Part B. 24 h post-seeding: application of cytotoxic compounds onto the cells

- Pre-warm the sterile phosphate buffered saline (PBS) and the sterile cell growth medium to 37 °C or room temperature
    - Volume of medium needed per one measurement plate: *ca* 16 mL in case if 100 μL volume per well is used
- Place under the laminar pipetting vessels, set of pipettes and suitable pipette tips, and one or more 96-well plates for dilutions of compounds
- Prepare the stock solutions of compounds of interest: it is advisable to make 1000-fold concentrated stocks (relative to the highest concentration that needs to be tested on cells) in the cell culture grade-dimethyl sulfoxide (DMSO)
    - N,N-dimethylformamide, ethanol, distilled water, or PBS can also be used for dissolving the compounds (dependent on the individual solubility of each tested compound)
    - If necessary, filter the compound stocks under the laminar using a sterile syringe and a suitable sterile filter (pore size 0.2-0.45 μm, hydrophobicity depends on the solvent used) to ensure the aseptic conditions
- Prepare dilutions of tested compounds in PBS on the 96-well plate
    - A maximum of six different compounds can be tested per one cell plate (in duplicates); for each compound, you can prepare one dilution series (a total of 5 different concentrations) that can be used for all replicates
    - Recommended volume per well: not less than 80 μL; the exact required volume (V) can be calculated according to the number of replicates (N) per plate: $V = 20\ \mu L \cdot N + 20\ \mu L$
    - In the example below, three compounds of interest are prepared for screening in duplicates in two cell lines:
        - To the wells in rows B–F in columns 1-3 of the dilution plate, add 100 μL of PBS
        - To the wells in row G in columns 1-3 of the dilution plate, add 198 μL of PBS
        - To well G1, add 2 μL of the compound X stock; to well G2, add 2 μL of the compound Y stock; to well G3, add 2 μL of the compound Z stock
        - Using the multichannel pipette, mix the contents of the wells in row G at least 10 times and transfer 20 μL to row F; mix again and continue the 6-fold dilutions until row C; do not transfer compound to row B, as this will be used as a non-treated control
- Using the multichannel pipette and a pipetting vessel, exchange medium over cells: add 180 μL of growth medium per well
- Using the multichannel pipette, transfer the dilutions of tested compounds from the dilution plate to the cell plate
    - For example:
        - Dilution series of compound X: take 20 μL of each dilution from column 1 of the dilution plate and transfer to columns 1, 4, 7, and 10 of the cell plate

    - Dilution series of compound Y: take 20 μL of each dilution from column 2 of the dilution plate and transfer to columns 2, 5, 8, and 11 of the cell plate
    - Dilution series of compound Z: take 20 μL of each dilution from column 3 of the dilution plate and transfer to columns 3, 6, 9, and 12 of the cell plate

An example layout at this stage in case if two cell lines are used (NT stands for non-treated and dil for the dilution):

	1	2	3	4	5	6	7	8	9	10	11	12
A												
B	NT	NT	NT	NT	NT	NT	NT	NT	NT	NT	NT	NT
C	X, dil 5	Y, dil 5	Z, dil 5	X, dil 5	Y, dil 5	Z, dil 5	X, dil 5	Y, dil 5	Z, dil 5	X, dil 5	Y, dil 5	Z, dil 5
D	X, dil 4	Y, dil 4	Z, dil 4	X, dil 4	Y, dil 4	Z, dil 4	X, dil 4	Y, dil 4	Z, dil 4	X, dil 4	Y, dil 4	Z, dil 4
E	X, dil 3	Y, dil 3	Z, dil 3	X, dil 3	Y, dil 3	Z, dil 3	X, dil 3	Y, dil 3	Z, dil 3	X, dil 3	Y, dil 3	Z, dil 3
F	X, dil 2	Y, dil 2	Z, dil 2	X, dil 2	Y, dil 2	Z, dil 2	X, dil 2	Y, dil 2	Z, dil 2	X, dil 2	Y, dil 2	Z, dil 2
G	X, dil 1	Y, dil 1	Z, dil 1	X, dil 1	Y, dil 1	Z, dil 1	X, dil 1	Y, dil 1	Z, dil 1	X, dil 1	Y, dil 1	Z, dil 1
H												
			↑ Cell line 1						↑ Cell line 2			

- Place the plate into a 37 °C CO_2 incubator and incubate under the usual cell growth conditions for 24 h or longer
    - Please note that the 24 h treatment time with the compounds may not be sufficient to bring forth the cytotoxic effect; hence, we recommend longer treatment times (48 h or 72 h)

<u>*Part C. 48 h post-seeding or later: resazurin assay*</u>

- Pre-warm the PBS supplemented with Ca^{2+} and Mg^{2+} to 37 °C or room temperature, switch on the microtiter plate reader, and prepare 2 pipetting vessels and a waste dish
    - If the machine enables the internal temperature control, set the incubator to 37 °C
- Prepare solution of resazurin in PBS into a centrifuge tube (50 μM)
    - Volume required: 10 mL per one measurement plate
    - In case of small-scale measurements, preparation of one or several more concentrated stock solutions (e.g., 100 mM and/or 5 mM resazurin in distilled water) might be advisable, from which the necessary dilution into PBS can be subsequently made
        - The stock solutions can be stored at -20 °C for a couple of months
- Define the measurement protocol
    - If the machine enables the kinetic measurement mode: perform measurement for 2 h with the reading(s) taken after each 15 min
    - If the machine enables measurement of both fluorescence intensity and absorbance: measure both physical quantities
        - Fluorescence intensity: excitation at 540 nm or similar, emission at 590 nm or similar
        - Absorbance: a 540-570 nm wavelength is suitable for monitoring formation of resorufin within the course of the reaction; a 600-620 nm wavelength is suitable for monitoring consumption of resazurin within the course of the reaction
- The following work does not have to be carried out under the aseptic conditions unless the experiment presupposes continuation of the live cell treatment following the resazurin assay and washout of the sensor
- Using the multichannel pipette, remove the cell culture medium from the plate with cells

- Using the multichannel pipette and the pipetting vessel, wash the cells briefly with PBS supplemented with Ca^{2+} and Mg^{2+} (100 μL per well) to remove the trances of growth medium
    - This step is not necessary if the phenol red-free medium was used post-seeding
- Using the multichannel pipette, discard the PBS washing solution and transfer the resazurin solution from the pipetting vessel onto the cells (100 μL per well)
- Remove water from 3 wells in row A and 3 wells in row B and add resazurin solution to these wells (no cells control)
- Place the plate into the reader and start the measurement immediately
    - If the microplate reader settings enable keeping the plate closed with lid, use the corresponding option; however, make sure that no condensate resulting from the temperature differences was formed on lid during the previous steps as the condensate may interfere with the readout signal
- For data analysis, pool the replicates and plot the XY graph picturing dependence of the readout on the logarithm of compound concentration tested
    - According to the given protocol, the highest concentration of the compounds used for treatment (row G) equals to 1000-fold dilution of the initial stock in DMSO
    - For each cell line, make a separate XY graph
    - For multiple readouts, make one XY graph for each type of readout
    - If the kinetic measurement mode is used, determine the last time-point that is still within the linear range of the assay and plot the data for this time-point only
    - Analyse the data in the graphs using the logarithmic dose–response curve to determine the $logIC_{50}$ values of the compounds of interest
        - You can also normalize the data by using signal from wells with non-treated cells as 100% viability control and signal from wells with resazurin but no cells as 0% viability control
        - If the signal measured for the cells treated with higher concentrations of the compound is significantly higher than that of the no-cell control, it is a sign of a cytostatic effect of the given compound of interest; in this case, the datapoint corresponding to the no-cell control should not be included into the dose–response curve

Reference

1. Riss, T.L.; Moravec, R.A.; Niles, A.L.; Duellman, S.; Benink, H.A.; Worzella, T.J.; Minor, L. Cell Viability Assays. In Assay Guidance Manual; Markossian, S., Grossman, A., Brimacombe, K., Arkin, M., Auld, D., Austin, C.P., Baell, J., Chung, T.D.Y., Coussens, N.P., Dahlin, J.L., et al., Eds.; Eli Lilly & Company and the National Center for Advancing Translational Sciences: Bethesda, MD, USA, 2004.
2. Roche: Colorimetric Assay (MTT Based) for the Nonradioactive Quantification of Cell Proliferation and Viability. Available online: https://www.sigmaaldrich.com/deepweb/assets/sigmaaldrich/product/documents/117/135/11465007001bul.pdf (accessed on 17 March 2022).
